# Vector-Borne and Zoonotic Diseases in the Eastern Mediterranean Region: A Systematic Review

**DOI:** 10.1007/s44197-023-00091-7

**Published:** 2023-02-09

**Authors:** Shaffi Fazaludeen Koya, Salma M. Abdalla, Chiori Kodama, Mory Keita, Abdinasir Abubakar

**Affiliations:** 1 School of Public Health, Boston University, Boston, USA; 2 World Health Organization Eastern Mediterranean Regional Office, Health Emergencies Programme, Cairo, Egypt

**Keywords:** Vector-borne diseases, Zoonosis, Infections, Incidence, Arboviral, Mosquito-borne diseases, Emerging infection, Epidemiology

## Abstract

**Background and Objective:**

World Health Organization Eastern Mediterranean Region (WHO EMR) has 40% people in the world in need of humanitarian assistance. This systematic review explores selected vector-borne and zoonotic diseases (VBZDs) of importance to EMR in terms of disease burden across countries and periods, disaggregated across sex, age groups, education levels, income status, and rural/urban areas, related vector or animal source reduction measures, and public health, social and economic impacts and related interventions.

**Methods:**

We used the Preferred Reporting Items for Systematic Reviews and Meta-Analyses (PRISMA) guidelines and reviewed articles in PubMed, Embase, and WHO Global Index Medicus published between 1st of January 2011 and 27th of June 2022. Thirteen VBZDs with at least one reported outbreak in the last five years in the region or prioritized as per previous analysis at the WHO global and regional level and based on expert consultations, were included as part of the analysis.

**Results:**

The review included 295 studies—55% on leishmaniasis and dengue combined, and 75% studies from Pakistan, Kingdom of Saudi Arabia, and Iran combined. Hospital-based and nationally representative studies constituted 60% and 10% respectively. Males were predominantly affected in most diseases; children reported high burden of Leishmaniasis, whereas elderly had a higher burden of Dengue Fever and Middle East Respiratory Syndrome. Although very few studies reported on socioeconomic differences in burden, the ones that reported showed higher burden of diseases among the disadvantaged socioeconomic groups such as the poor and the less educated. More than 80% studies reported an increase in burden over the years.

**Conclusion:**

The literature is scanty for most of the diseases reviewed and the number of studies from countries with humanitarian challenges is very low. The need for more nationally representative, population-based studies calls for prioritizing research investments.

**Supplementary Information:**

The online version contains supplementary material available at 10.1007/s44197-023-00091-7.

## Introduction

Globally vector-borne and zoonotic diseases (VBZDs) lead to around 10 million deaths every year. [1, 2] The socioeconomically underprivileged are particularly affected by VBZDs, such as leishmaniasis, dengue fever (DF), and malaria, as they have high morbidity and mortality rates [3, 4]. The complexity of epidemiology linked to various social and economic interactions of these diseases makes it challenging to assess the social, economic, and health system-related costs of VBZDs, which makes formulating policies and allocating resources challenging [4].

The World Health Organization (WHO) Eastern Mediterranean Region (EMR) [5, 6] suffers a disproportionate share of the global burden of VBZDs [7]. The well-developed international trade with neighboring countries, unplanned urbanization, and the close proximity of animal rearing with human settlements in rural areas make the EMR particularly vulnerable to zoonotic illnesses and frequent outbreaks of emerging infectious diseases [8]. These outbreaks have primarily been sparked by the varying surveillance levels and cooperative capacities of the countries at the animal health–human health interface [1, 9]. Additionally, the region is home to almost 40% of all people around the world in need of humanitarian assistance [6], challenged by fragile health systems, and suboptimal disease surveillance, preparedness, and response capacities [10].

The burden of emerging VBZDs, risk factors, geographic distribution, seasonal trends and public health, and societal impact remains unknown despite the significant impact on the health of population and economic development in region. A systematic review can help to prioritize VBZDs through careful analysis such that targeted interventions including proven prevention and control strategies can be delivered.

### Objective

This review aimed to understand the published literature from EMR countries, describe the epidemiology particularly the disease burden, across countries and periods, disaggregated across sex, age groups, education levels, income status, and rural/urban areas, and narrate the related vector or animal source reduction measures, and public health, social and economic impacts and related interventions. Specifically, we aimed to answer the following research questions:

1. Does the burden of EMR relevant VBZDs vary across countries?
2. Does the burden vary across age groups, sex, education levels, income status, and geographies (rural vs urban)? Does the burden change over time?
3. What does the literature talk about the public health measures to control these diseases including vector or animal source reduction measures?
4. What are the social and economic impacts of these VBZDs reported in literature?

## Methods

We conducted the review following the preferred reporting items for systematic reviews and meta-analyses (PRISMA) guidelines. All member states in the EMR (Supplement, S1) were included in the review and we considered all articles published in English and Arabic languages from 1 January 2011. The final search was conducted on 27 June 2022.

### Sources and Criteria

We conducted systematic literature searches of three databases—PubMed, Embase, and WHO Global Index Medicus. The diseases included in the review were selected on a two stage process. In the first stage, we included all relevant VBZDs that met the following criteria:

1. At least one reported outbreak in the last five years in any of the countries as per WHO EMR Office data [11], or
2. Diseases prioritized as per previous analysis at the WHO global [1] and regional level [12].

This initial list had 16 diseases: Chikungunya, Coronavirus disease of 2019 (COVID-19), DF, Yellow Fever (YF), Zika, Crimean–Congo hemorrhagic Fever (CCHF), Zoonotic Influenza A (avian, swine and other zoonotic influenza viruses), Leishmaniasis, Leptospirosis, Middle East Respiratory Syndrome (MERS), Monkeypox, Plague (*Yersinia pestis*), Rabies, Rift Valley Fever (RVF), Severe acute respiratory syndrome (SARS), and West Nile Fever (WNF). From this list, based on expert consultation, we excluded COVID-19 and Monkeypox as these diseases are already prioritized as they are diseases of Public Health Emergency of International Concern. SARS was excluded as there were no reported cases in the region. Ultimately, the review considered the remaining 13 diseases.

### Search Strategy

Initially we searched PubMed using MeSH and non-MeSH terms. The search strings had three components: country names, disease names, and epidemiological characteristics. Then we extended the searches to Embase and WHO Global Index Medicus databases using similar search strings. (Supplement S2) We conducted additional searches to identify papers that discussed social or economic impact of VBZDs and papers that discussed public health measures. (Supplement S3–S4).

### Screening and Selection

All the papers extracted through the search in PubMed, Embase, and Global Index Medicus were pooled and screened (title and abstract) using Rayyan, an online software program for systematic reviews [13]. First, we removed duplicates of the articles across three databases, and then one researcher conducted title and abstract screening. The screened-in articles were downloaded in a spreadsheet and two independent reviewers read each of the full papers to ascertain the eligibility to include in the review. In the final step, data on pre-determined relevant variables were extracted from the included papers using a data extraction sheet.

### Exclusion Criteria

We excluded systematic reviews, conference papers, narrative reviews, editorials, and correspondences without any empirical data. Studies that primarily discussed countries outside of EMR with passive reference to EMR countries and studies that passively referenced the listed diseases were excluded.

### Data Extraction

Two authors collaboratively reviewed and extracted the following relevant information from each paper: authors, published year, study year, country, incidence/prevalence measures (disaggregated across sex and rural/urban areas), mortality rates (disaggregated numbers, and percentages across sex), and reported difference in incidence/prevalence/mortality rates across sex, age groups, rural/urban, education levels, and income status. (Supplement S5) Disagreements between reviewers were sorted out through discussions and pending discrepancies were resolved by the lead reviewer.

### Analysis

First, we described the study characteristics using numbers and proportions of studies across characteristics. Second, we described the incidence/prevalence of diseases as reported by authors and compared across countries for each disease including reported differences across sex, geography (rural/urban), age groups, time, and socioeconomic groups. Third, we described the reported differences in disease-specific mortality rates across countries by sex, age groups, geography (rural/urban), and time. Significant differences as reported by the authors using *p* values or 95% confidence intervals were used to determine differences across groups. Fourth, we extracted the seroprevalence measures reported by individual studies and calculated the median and interquartile range (IQR) for each disease. We identified the sample size (denominator) and test positives (numerator) wherever possible and calculated the rate ourselves. Lastly, we summarized the response measures in terms of testing and treatment services availability, vector burden, overall disease control, overall vector control, overall economic impact, and overall public health measure as ascertained by the authors in the papers and reviewed by the reviewers. We conducted all the analysis including creating the maps using Microsoft Excel version 16.6 and R software version 4.1.1 (R Core Team, 2020).

## Results

**Fig. 1:**
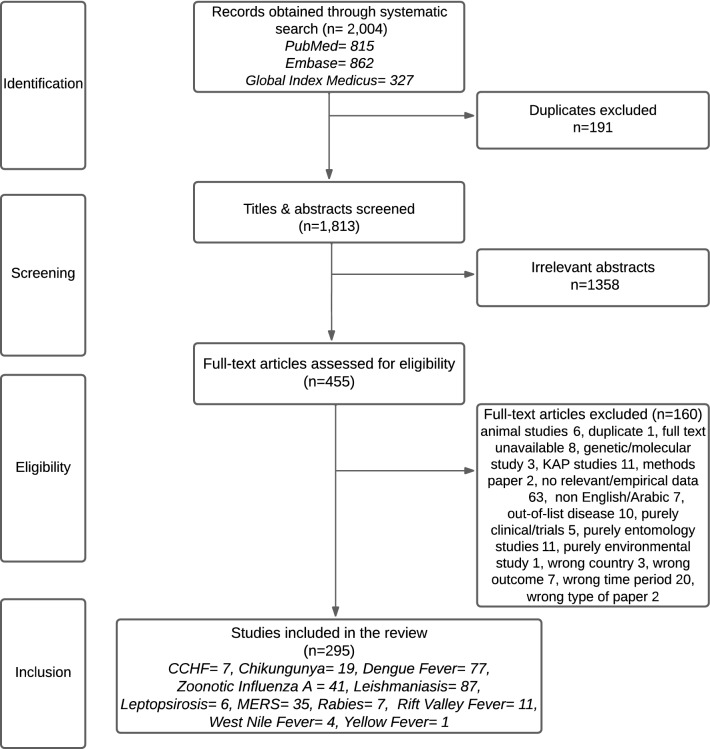
PRISMA flowchart—review of vector-borne and zoonotic diseases in the Eastern Mediterranean region. *CCHF* Crimean–Congo hemorrhagic Fever, *MERS* Middle East Respiratory Syndrome

We found 2004 papers across PubMed, Embase, and Global Index Medicus, and after deduplication, we screened 1813 papers. (Fig. 1) The final review included 295 studies, with the earliest study containing data from 2008. (Supplement S6–S9) Leishmaniasis (*N* = 87) and DF (*N* = 77) had the highest number of publications. Very few studies were available of CCHF (*N* = 7), Rabies (*N* = 7), Leptospirosis (*N* = 6) and WNF (*N* = 4). There was one paper on YF, while no relevant papers were found on Plague or Zika after the final screening. Three nations accounted for over three-fourths of the papers: Iran (22%), Pakistan (25%), and the Kingdom of Saudi Arabia (KSA) (23%), and no papers were based on data from Bahrain, Kuwait, Occupied Palestinian territory, and Somalia.

The bulk of studies was cross-sectional in nature (*N* = 239, 81%), including multi-year cross-sectional studies (*N* = 110, 37%). (Table 1) 9.5% of studies had data that were nationally representative, while the majority (63%) used hospital data and 30% used data at the population level. Most studies (77%) employed primary data, while 18% of studies (*N* = 52) were based on outbreak investigations. Most studies had data on all age groups (74%) and both sexes (86%).

**Table 1:** Study characteristics—review of vector-borne and zoonotic diseases in the Eastern Mediterranean region

Characteristics	Number of studies *N* = 295^1^
Study design	
Cross-sectional	239 (81%)
Cohort	22 (7.5%)
Other designs	19 (6.4%)
Case control	12 (4.1%)
Longitudinal study	2 (0.7%)
Interventional study	1 (0.3%)
Study setting	
Hospital-based	186 (63%)
Community-based	89 (30%)
Mixed	20 (6.8%)
Data level	
Subnational	267 (91%)
National	28 (9.5%)
Data source	
Primary data	227 (77%)
Secondary data	51 (17%)
Both primary and secondary data	15 (5.1%)
Not available/applicable	2 (0.7%)
Outbreak investigation	52 (18%)

### Disease-Specific Findings

#### Crimean–Congo Hemorrhagic Fever (CCHF)

All the seven studies from five countries—Afghanistan, Iran (*n* = 3), Oman, Pakistan, and Sudan—were cross-sectional. Six studies were community-based, subnational, and used primary data, and one study reported time trend. Two studies (29%) found higher incidence and one study each reported higher mortality among males compared to females, and among young adults. Six studies did not provide data on differences in incidence by socio-economic status and no study reported differences by geography. One study reported good vector control measures while the rest did not provide data on vector control, and no study provided relevant data on mortality differences by time or geography.

#### Chikungunya

There were 19 studies on Chikungunya drawing on data from seven countries, highest from Pakistan. Studies were primarily hospital-based (68%), and all studies had data at subnational level. Studies showed an increasing trend in burden over time with higher incidence in males reported and in urban areas. 18 (95%) studies reported high vector burden, and twelve studies (63%) reported poor vector control measures while six (32%) reported average vector control. Two studies reported data on differences in burden by socio-economic status and three studies reported differences by geography. 79% studies reported poor disease control measures. Three studies showed higher mortality among males conversely to one study among females. In general, more studies reported higher mortality among the elderly (12%) and more studies reported increasing incidence trends.

#### Dengue Fever (DF)

Most of the 77 studies on DF were hospital-based (71%) and subnational (94%), and studies reported higher burden of DF in urban areas. 62% of studies did not assess time trends. Most studies (57%) found a higher burden among males compared to females, and among the adults (82%) except that elderly reported higher burden in Pakistan, Saudi Arabia, and Sudan. At least 41% studies reported average to poor vector control, and 75% studies reported average to poor disease control in general. 81% studies did not provide data on differences in burden by socio-economic status and 74% did not provide differences in burden by geography although 21% reported a higher burden in urban areas compared to rural areas. The majority of studies did not provide data on morality.

#### Zoonotic Influenza A

Of the 41 studies, 66% were hospital-based, 80% used subnational data, and 27% had a cohort design. 12% studies reported an increase in burden with time, 44% (*n* = 18) showed a higher burden among males, and 34% of studies reported disease burden was highest among adults while 24% reported highest among children. 29% of studies reported poor disease control overall. Most studies (80%) did not provide data on trends over time while 12% reported an increase in burden with time. Most studies did not provide data on differences in burden by geography (90%) or socio-economic status (95%), or on mortality, except that 12% of studies reported a higher mortality rate among the elderly.

#### Leishmaniasis

Studies (*n* = 87) showed a comparable divide between hospital-based (54%) and community-based (43%). 97% studies used subnational data and solely used primary data (83%). While 18% studies showed an increase in burden with time, another 10% showed a decrease. Thirty-nine studies (45%) showed a higher burden among males. The burden was highest among children in 44% of studies. While 21% studies reported higher burden among rural areas, 13% reported higher burden in urban areas, and 14% studies showed differences by socio-economic status. Although 34% studies reported high vector control, 11% studies reported poor vector control. Most studies did not provide adequate data on mortality.

#### Leptospirosis

Of the six studies, four studies were multi-year cross-sectional; three were hospital-based, and all studies had subnational data. Three studies showed a higher burden of disease among males, and two studies reported a higher burden in rural areas. None of the studies provided data on differences in burden by socio-economic status, data on differences in mortality based on time, age, sex, or geography, data on vector control or disease control, or time trends in disease burden.

#### Middle East Respiratory Syndrome (MERS)

All the 35 studies on MERS came from three countries—Qatar, KSA, and United Arab Emirates, mostly from KSA (91%). 71% of studies were cross-sectional, hospital-based (74%) and used subnational data (77%). Equal number of studies (69% each) showed a higher incidence in adults and men. None of the studies provided data on time trends or on differences in incidence by geography or socio-economic status. 49% studies reported higher mortality among males, and 46% reported a higher mortality burden among the elderly. Most studies (97%) did not provide data on mortality rate by geography, while no study provided data on differences in mortality over time.

#### Rabies

The seven studies drew data from five countries—Iran, Oman, Pakistan, Tunisia, and Yemen, of which three were multi-year cross-sectional, and two studies were longitudinal. Most studies were hospital-based (71%), used subnational secondary data (57%). Two studies reported higher burden among adults and males, while one reported higher burden among children and among females. No studies provided data trends by time, geography, or socio-economic status. None of the studies reported on host animal control. Equal number (1 each) studies reported increase, decrease, and no significant changes in mortality across time.

#### Rift Valley Fever (RVF)

The 11 studies came four countries—Djibouti, Egypt, KSA, and Sudan. The studies were a mix of community-based (55%) and hospital-based (45%). All studies assessed subnational data. Most studies were based solely on primary data (82%). None of the studies provided data trends by time or urban–rural differences. Two papers each reported higher burden among children and among males compared to one which reported higher burden among adults and none among females. No papers provided data on mortality differentials. A very high vector burden was reported by 10 studies (91%). 27% studies reported good vector and disease control.

#### West Nile Fever (WNF)

There were four studies on WNF, drawing on data from three countries—Iran, Pakistan, and Tunisia. All studies were multi-year cross-sectional, hospital-based, used subnational data. Two showed an increased incidence over time, while none of the studies provided data by either geographic differences, socio-economic status, or on morality by age, time, sex, or geography, or on vector control.

#### Yellow Fever (YF)

The single study published in 2013 from Sudan—which was cross-sectional, community-based, using primary data—reported a higher incidence among males and in rural areas. The study reported differences across socio-economic groups, showed a higher mortality among males and among young adults. The study did not provide any time trend.

### Seroprevalence Measures

The median seroprevalence was 21.8% (IQR—10.4%–29.7%) for DF, 18.7% for cutaneous leishmaniasis (IQR—11.9%–52.1%), and 11.8% (IQR—6.7%–19.5%) for CCHF. (Table 2, Supplement S8).

**Table 2:** Seroprevalence rates of vector-borne and zoonotic diseases in the Eastern Mediterranean region

Disease	No. of studies	Median seroprevalence	Interquartile range
Dengue fever	15	21.8%	10.4%–29.7%
Cutaneous leishmaniasis	9	18.7%	11.9%–52.1%
Visceral leishmaniasis	6	1.5%	0.7%–3.2%
Zoonotic influenza A	7	11.5%	5.8%–18.1%
CCHF	4	11.8%	6.7%–19.5%
Chikungunya	3	2.6%	2.6%–13.9%
Rift valley fever	3	2.2%	1.1%–6.7%
Leptospirosis	1	20.00%	–
MERS	1	17.10%	–
Yellow fever	1	25.00%	–
West Nile fever	1	0.60%	–

*CCHF* Crimean–Congo hemorrhagic Fever, *MERS* Middle East Respiratory Syndrome

### Sex and Age Differences

Males were predominantly affected by all diseases studied—144 studies reported higher morbidity among males compared to 23 among females. However, we saw a substantial number of papers reporting higher morbidity among females in Leishmaniasis in Iran. Mortality was reported higher among males for most diseases by most studies; however, one study each reported higher mortality for females in DF (Pakistan) and Rabies (Oman) and two studies in MERS (KSA), while no difference in mortality was reported by two papers each in DF and MERS.

176 studies reported significant difference across age groups in reported burden of disease. Adults shared the highest burden for all diseases except WNF, RVF, and Leishmaniasis. Children reported very high burden in Leishmaniasis in Iran. The elderly reported higher burden in DF in Pakistan, KSA, and Sudan, and in MERS in KSA. 37 studies (12.5%) reported differences in mortality across age groups, and of these, two reported no significant differences. Significant differences were reported in CCHF, DF, Zoonotic Influenza A, Leishmaniasis, MERS and YF. Most studies (*n* = 25) reported highest mortality among the elderly including all 16 studies on MERS which reported data. Exceptions were for DF in Pakistan and Sudan, were one study each reported higher mortality among children and two studies from KSA which reported higher mortality among children in Zoonotic Influenza A. Similarly, one study on CCHF from Afghanistan, one study on YF from Sudan and three studies on DF from Pakistan reported higher mortality among young adults.

### Time Trends and Urban–Rural Differences

Most studies did not report data on time trends changes (*n* = 214) over years. Among those that reported time trends (*n* = 81), the majority (80%) reported increasing burden of diseases over time. Similarly, 80% studies did not have urban–rural stratified data. Among studies with data available, similar proportion of studies reported higher morbidity in rural (42%, *n* = 26) and urban (49%, *n* = 30) areas. The urban rural differences in burden were mostly pronounced in DF (80% studies reported higher burden in urban areas) and Leishmaniasis (60% studies reported higher burden in rural areas). CCHF, MERS, RVF, and WNF studies did not report data on rural–urban differences.

### Socioeconomic Differences

Very few (*n* = 41, 14%) studies reported data on difference in morbidity across socioeconomic groups. Leptospirosis, MERS, and WNF studies did not report any data on socioeconomic differences. Among those that had data available, 28 studies reported significant differences across at least one feature characteristic of socioeconomic stratification like income, education, or social class. Among these 28 studies, 24 studies reported that disadvantaged groups had higher burden of diseases, particularly DF and Leishmaniasis.

### Testing and Treatment Availability

Overall, 83 studies reported data on disease testing availability and treatment availability. Studies with data on testing were mostly from Pakistan (*n* = 23) and Iran (*n* = 21). The maximum number of studies that reported availability of testing was on Zoonotic Influenza A (*n* = 30). Studies with data on treatment facility were mostly from Pakistan (*n* = 31), KSA (*n* = 13), and Iran (*n* = 12). Most studies on Zoonotic Influenza A (70%) reported good testing availability, while 22% reported poor treatment availability. Most studies on Leptospirosis (75%) reported good testing availability while no study reported data on treatment availability. The one study on YF reported poor testing and treatment availability.

**Fig. 2:**
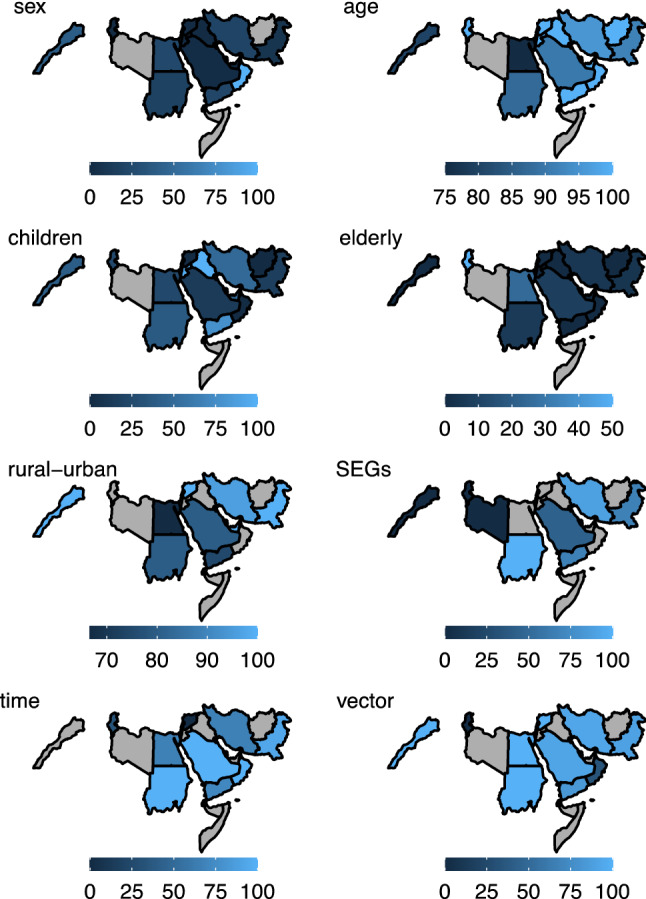
Proportion of papers that reported differences in key epidemiological features of vector-borne and zoonotic diseases in the Eastern Mediterranean region. Note: the map (1, sex) refers to proportion of papers that reported higher morbidity among female, (2, age) shows proportion of papers that reported significant differences across age groups, (3, children) shows proportion of papers that reported significantly higher morbidity among children, (4, elderly) shows proportion of papers that reported significantly higher morbidity among elderly, (5, rural–urban) shows proportion of papers that reported significant differences across rural and urban areas, (6, SEGs) shows proportion of papers that reported significantly higher burden among poor socioeconomic groups (SEGs), (7, time) shows proportion of papers that reported significant increase in incidence/prevalence over time, and (8, vector) shows proportion of papers that reported significantly high vector density among papers that had data for these variables. Maps were created using R software ggplot2 package. Light gray indicates no data were reported

Countries showed some important differences in the epidemiological features of the diseases in general. (Fig. 2) Iran reported a far greater number of studies with higher morbidity among females and children, while KSA reported more studies with higher morbidity among elderly. Iran and Pakistan reported significant differences between urban and rural areas and socioeconomic groups. More studies from Pakistan and Iran showed increase in disease burden over time.

### Social and Economic Cost

Very few studies examined the socio-economic cost of VBZDs in the region. Studies showed that economic costs in the broiler farm and camel trade industries were devastating unless surveillance strategies were improved and the time interval between disease identification and stamping out was reduced [14, 15]. In addition, studies showed significant out-of-pocket expenditure for households and direct and indirect effects on society regarding lost productive years, livelihood, and jobs [16, 17]. In Sudan, a study by Meheus et al. reported that despite free provisions of Visceral Leishmaniasis (VL) drugs at public hospitals, more than 75% of households incurred catastrophic out-of-pocket expenditures—close to 40% of annual household income for treating just one VL episode [16].

### Public Health Interventions

**Fig. 3:**
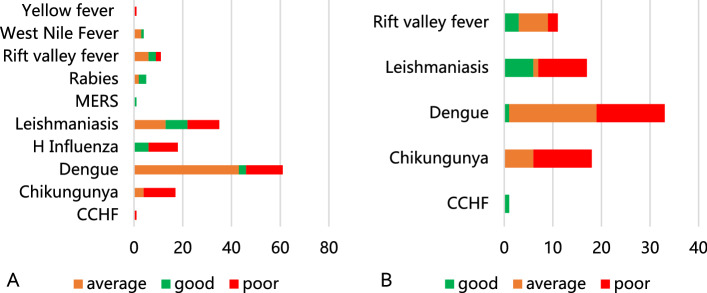
Vector-borne and zoonotic diseases in the Eastern Mediterranean region—percentage of studies reporting varying levels of: **A** overall disease control measures, and **B** animal source/vector control measures. Note: the percentage of studies is calculated out of number of papers that had the data on these measures for specific diseases; diseases which did not have papers with relevant data are not shown. *CCHF* Crimean–Congo hemorrhagic Fever, *MERS* Middle East Respiratory Syndrome

Most studies included in the systematic review reported poor prevention and control measures. Among studies with data, one-fourth reported good overall disease control for Leishmaniasis, RVF, and WNF. DF had the lowest proportion of studies that reported good overall disease control. (Fig. 3) Animal source/vector control measures were reported poor by most studies on Chikungunya, DF, and Leishmaniasis.

A strong routine surveillance complemented by immediate response measures [18], and case management guidelines were effective in controlling outbreaks in Oman [19]. A 2020 study [20] from Pakistan demonstrated the utility of big data analytics and pointed to the need for localized containment activities and improved allocation of resources. Community mobilization and active source reduction can assist to prevent, control and break the transmission cycle in outbreaks. Study from Sudan [21] demonstrated the effectiveness of evidence-based interventions in DF control. The short-memory around disease prevention measures [22] suggested the need for contextually relevant campaigns alongside the efforts of local municipal and federal governments.

## Discussion

Our systematic review of multiple VBZDs in EMR showed substantial burden of VBZDs in the region with significant differences between countries in terms of availability of data. Besides, we found scarcity of data for several countries with humanitarian challenges. Even with limited data on the determinants of these VBZDs, we observed profound differences in burden across socioeconomic groups.

We detected an increasing trend for most of the diseases. All studies with data on time trends from Qatar, Oman, Sudan, and KSA, and more than two-third studies from Pakistan, Yemen, Egypt, and Iran reported increasing burden of disease over the years. All studies from Djibouti, Morocco, Qatar, Sudan, Egypt, and Syrian Arab Republic, and more than 80% studies from Iran, KSA, Pakistan, and Yemen reported high vector burden.

There is scarcity of data on several diseases examined in many countries, especially countries faced with humanitarian challenges. A previous review focused on WNF epidemiology in the region [23]. Our review did not find any study from Palestine or Somalia, while we found one study each from Afghanistan, Libya, Syria, and Iraq, and 11 studies from Yemen. Further, we found one study on YF, four studies on WNF, and six studies on Leptospirosis. Our review, however, also revealed several comprehensive studies—including those on MERS from KSA [24], DF from Pakistan [25, 26], and CCHF from Sudan [27].

Lastly, we noted significant gender and socioeconomic inequities in the burden of diseases. More than 20% studies from Oman, Morocco, Egypt, Yemen, and Iran reported higher morbidity among females. More than 30% studies from Djibouti, Iraq, Jordan, Yemen, Qatar, United Arab Emirates, and Iran reported higher morbidity among children. Studies from Tunisia, Egypt, KSA, Sudan, Iran, and Pakistan reported higher morbidity among elderly. Most studies with data from Pakistan, United Arab Emirates, Morocco, Syrian Arab Republic, Iran, KSA, Sudan, Yemen, and Egypt showed significant rural–urban differences in disease burden. Significant differences in disease burden across socioeconomic groups were reported from Djibouti, Sudan, Iran, Pakistan, and Yemen; and in most cases adversely affecting the poor and the less educated.

Our review has some limitations. First, this review did not include all known VBZDs. However, our objective was to assess the key VBZDs in the region based on available data through outbreak investigations and national surveillance systems. Second, our review was limited to studies in the last 11 years as we wanted to get a recent overview. Third, as we included only three databases, we might have missed some articles in other databases. Lastly, we did not investigate the quality of studies included in our review.

The review reiterates the importance of multi-sectoral, transdisciplinary approach that recognizes the interconnection between the health of people, animals, and their shared environment in prevention and control of VBZDs in the region. More specifically, the region needs to identify strategies to strengthen the collaboration and information sharing of surveillance and laboratories among human and animal sectors, identify best practices across multidisciplinary team to enhance joint response by human and animal sectors, and set up research priorities.

## Conclusion

Literature on VBZDs in EMR is scarce, especially in countries with humanitarian challenges. To gain a deeper understanding of the epidemiology and socioeconomic impact of VBZDs, we need to improve disease surveillance systems and develop nationally representative, population-based studies.

## Supplementary Information

Below is the link to the electronic supplementary material.

Supplementary file1 (PDF 483 KB)

## Data Availability

All data related to this paper are provided within the main paper and the supplement files.

## Abbreviations

COVID-19: Coronavirus disease of 2019
CCHF: Crimean–Congo hemorrhagic Fever
DF: Dengue fever
EMR: Eastern Mediterranean Region
KSA: Kingdom of Saudi Arabia
MERS: Middle East Respiratory Syndrome
PRISMA: Preferred reporting items for systematic reviews and meta-analyses
RVF: Rift Valley fever
SARS: Severe acute respiratory syndrome
VBZDs: Vector-borne and zoonotic diseases
UAE: United Arab Emirates
WNF: West Nile fever
WHO: World Health Organization
YF: Yellow fever
