# Supplementary material for: Vector-Borne and Zoonotic Diseases in the Eastern Mediterranean Region: A Systematic Review
Source: J Epidemiol Glob Health. 2023 Feb 9;13(1):105–14. doi: 10.1007/s44197-023-00091-7 (PMC9910263; doi:10.1007/s44197-023-00091-7)
Supplement: Supplementary file 1 — Supplementary file1 (PDF 483 KB) [file 44197_2023_91_MOESM1_ESM.pdf]

Vector-Borne And Zoonotic Diseases In The Eastern Mediterranean Region: A Systematic Review

Shaffi Fazaludeen Koya, Salma M Abdallah, Chiori Kodama et al.

Supplement files

S1: Countries in WHO EMR

Afghanistan, Bahrain, Djibouti, Egypt, Iran (Islamic Republic of), Iraq, Jordan, Kuwait, Lebanon, Libya, Morocco, Occupied Palestinian territory, Oman, Pakistan, Qatar, Saudi Arabia, Somalia, Sudan, Syrian Arab Republic, Tunisia, United Arab Emirates, and Yemen

S2: Search strings

Strings used for the main search —Epidemiological features of VBZDs

Names of countries

("Afghanistan"[Mesh] OR “Afghanistan” OR "Bahrain"[Mesh] OR “Bahrain” OR "Djibouti"[Mesh] OR “Djibouti” OR “Somaliland, French” OR “Republic of Djibouti” OR “French Somaliland” OR "Egypt"[Mesh] OR "Arab Republic of Egypt" OR "United Arab Republic" OR "Iran"[Mesh] OR “Iran” OR “Islamic Republic of Iran” OR "Iraq"[Mesh] OR “Iraq” OR “Republic of Iraq” OR "Jordan"[Mesh] OR “Jordan” OR "Kuwait"[Mesh] OR “Kuwait” OR "Lebanon"[Mesh] OR “Lebanon” OR “Lebanese Republic” OR "Libya"[Mesh] OR “Libya” OR "Morocco"[Mesh] OR “Morocco” OR “Ifni” OR "Oman"[Mesh] OR “Oman” OR “Muscat” OR "Pakistan"[Mesh] OR “Pakistan” OR “Islamic Republic of Pakistan” OR “State of Palestine” OR “Occupied Palestinian Territory” OR "Qatar"[Mesh] OR “Qatar” OR “Katar” OR “State of Qatar” OR “Quatar” OR "Saudi Arabia"[Mesh] OR “Saudi Arabia” OR “Kingdom of Saudi Arabia” OR "Somalia"[Mesh] OR “Somalia” OR "Sudan"[Mesh] OR “Sudan” OR “Republic of the Sudan” OR "Syria"[Mesh] OR “Syria” OR “Syrian Arab Republic” OR "Tunisia"[Mesh] OR “Tunisia” OR "United Arab Emirates"[Mesh] OR “United Arab Emirates” OR “Trucial States” OR “Abu Dhabi” OR "Yemen"[Mesh] OR “Yemen” OR “Republic of Yemen” OR “Democratic Yemen” OR “Sanaa” OR “North Yemen” OR “Aden” OR “South Yemen”)

Priority vector-borne and zoonotic diseases

- Dengue fever : "Dengue"[Mesh] OR “Dengue” OR “Breakbone Fever” OR “Fever, Breakbone” OR “Classical Dengue Fever” OR “Classical Dengue Fevers” OR “Dengue Fever, Classical” OR “Break-Bone Fever” OR “Break Bone Fever” OR “Fever, Break-Bone” OR “Dengue Fever” OR “Fever, Dengue” OR “Classical Dengue” OR “Classical Dengues” OR “Dengue, Classical”

- Chikungunya: "Chikungunya Fever"[Mesh] OR "Chikungunya Fevers" OR "Fever, Chikungunya" OR "Chikungunya Virus Infection" OR "Chikungunya Virus Infections" OR "Infection, Chikungunya Virus" OR "Chickungunya Fever" OR "Chickungunya Fevers" OR "Fever, Chickungunya"

- Yellow fever : "Yellow Fever"[Mesh] OR "Fever, Yellow" OR "Fevers, Yellow" OR "Yellow Fevers"

- Zika : "Zika Virus"[Mesh] OR "ZikV" OR "Virus, Zika" OR "Zika Virus Infection"[Mesh] OR "Infection, Zika Virus" OR "Virus Infection, Zika" OR "ZikV Infection" OR "Infection, ZikV" OR "Fever, Zika" OR "Zika Virus Disease" OR "Disease, Zika Virus" OR "Virus Disease, Zika" OR "Zika Fever" OR "Congenital Zika Syndrome" OR "Congenital Zika Virus Infection"

- Crimean-Congo haemorrhagic fever (CCHF): "Hemorrhagic Fever, Crimean"[Mesh] OR "Crimean Hemorrhagic Fever" OR "Fever, Crimean Hemorrhagic" OR "Congo Virus Infection" OR "Congo-Crimean Hemorrhagic Fever" OR "Congo Crimean Hemorrhagic Fever" OR "Fever, Congo-Crimean Hemorrhagic" OR "Hemorrhagic Fever, Congo-Crimean" OR "Infection, Congo Virus" OR "Crimean-Congo Hemorrhagic Fever" OR "Crimean Congo Hemorrhagic Fever" OR "Fever, Crimean-Congo Hemorrhagic" OR "-Congo" OR "Fever, Crimean-Congo Haemorrhagic" OR "Haemorrhagic Fever, Crimean-Congo"

- Influenza (avian, swine and other zoonotic influenza viruses): "Influenza, Human"[Mesh] OR "Human Influenzas" OR "Influenzas, Human" OR "Influenza" OR "Influenzas" OR "Human Flu" OR "Flu, Human" OR "Human Influenza" OR "Influenza in Humans" OR "Influenza in Human" OR "Grippe" OR "Orthomyxoviridae"[Mesh] OR "Orthomyxoviruses" OR "Orthomyxovirus" OR "Influenza Viruses" OR "Influenza Virus" OR "Myxoviruses" OR "Myxovirus"

- Leishmaniasis: "Leishmaniasis"[Mesh] OR "Leishmaniases" OR "Leishmania Infection" OR "Infection, Leishmania" OR "Infections, Leishmania" OR "Leishmania Infections"

- Leptospirosis: "Leptospirosis"[Mesh] OR "Leptospiroses" OR "Leptospira Infection" OR "Infection, Leptospira" OR "Infections, Leptospira" OR "Leptospira Infections" OR "Stuttgart Disease" OR "Mud Fever" OR "Fever, Mud" OR "Rice-Field Fever" OR "Fever, Rice-Field" OR "Rice Field Fever" OR "Cane-Cutter Fever" OR "Cane Cutter Fever" OR "Fevers, Cane-Cutter" OR "Swineherds Disease" OR "Swineherds Diseases" OR "Canicola Fever" OR "Fever, Canicola" OR "Leptospira Canicola Infection" OR "Infection, Leptospira Canicola" OR "Infections, Leptospira Canicola" OR "Leptospira Canicola Infections" OR "Leptospirosis Canicola" OR "Leptospirosis Canicolas"

- Middle East respiratory syndrome (MERS): "Middle East Respiratory Syndrome Coronavirus"[Mesh] OR "Middle East respiratory syndrome-related coronavirus" OR "Middle East respiratory syndrome related coronavirus" OR "MERS-CoV" OR "MERS Virus" OR "MERS Viruses" OR "Virus, MERS" OR "Viruses, MERS"

- Plague (Yersinia pestis): "Plague"[Mesh] OR "Yersinia pestis Infection" OR "Septicemic Plague" OR "Black Death" OR "Black Plague" OR "Pneumonic Plague" OR "Pulmonic Plague" OR "Bubonic Plague" OR "Meningeal Plague" OR "Yersinia pestis"[Mesh] OR "Pestisella pestis" OR "Bacterium pestis" OR "Yersinia pseudotuberculosis subsp. pestis" OR "Bacillus pestis" OR "Pasteurella pestis"

- Rabies: "Rabies"[Mesh] OR "Lyssa" OR "Lyssas" OR "Hydrophobia" OR "Rabies virus"[Mesh] OR "Rabies viruses"

- Rift Valley fever (RVF) : "Rift Valley Fever"[Mesh] OR "Fever, Rift Valley" OR "Rift Valley fever virus"[Mesh] OR “River Valley fever virus”

- Severe acute respiratory syndrome (SARS): "Severe Acute Respiratory Syndrome"[Mesh] OR "Respiratory Syndrome, Severe Acute" OR "SARS (Severe Acute Respiratory Syndrome)" OR "Respiratory Syndrome, Acute, Severe" OR "SARS Virus"[Mesh] OR "Severe Acute Respiratory Syndrome Virus" OR "SARS-Related Coronavirus" OR "Coronavirus, SARS-Related" OR "SARS Related Coronavirus" OR "SARS-CoV" OR "Urbani SARS-Associated Coronavirus" OR "Coronavirus, Urbani SARS-Associated" OR "SARS-Associated Coronavirus, Urbani" OR "Urbani SARS Associated Coronavirus" OR "SARS Coronavirus" OR "Coronavirus, SARS" OR "Severe acute respiratory syndrome-related coronavirus" OR "Severe acute respiratory syndrome related coronavirus" OR "SARS-Associated Coronavirus" OR "Coronavirus, SARS-Associated" OR "SARS Associated Coronavirus"

- West Nile virus (WNV): "West Nile virus"[Mesh] OR "Egypt 101 virus" OR "Kunjin virus" OR "West Nile Fever"[Mesh] OR "West Nile Virus Infection" OR "WNV Infection" OR "Infection, WNV" OR "Infections,

WNV" OR "WNV Infections" OR "West Nile Fever Myelitis" OR "West Nile Fever Meningoencephalitis" OR "Encephalitis, West Nile Fever" OR "West Nile Fever Encephalitis" OR "West Nile Fever Meningitis"

String to restrict to non-COVID studies

"COVID-19"[Mesh] OR "COVID 19" OR "SARS-CoV-2 Infection" OR "Infection, SARS-CoV-2" OR "SARS CoV 2 Infection" OR "SARS-CoV-2 Infections" OR "2019 Novel Coronavirus Disease" OR "2019 Novel Coronavirus Infection" OR "2019-nCoV Disease" OR "2019 nCoV Disease" OR "2019-nCoV Diseases" OR "Disease, 2019-nCoV" OR "COVID-19 Virus Infection" OR "COVID 19 Virus Infection" OR "COVID-19 Virus Infections" OR "Infection, COVID-19 Virus" OR "Virus Infection, COVID-19" OR "Coronavirus Disease 2019" OR "Disease 2019, Coronavirus" OR "Coronavirus Disease-19" OR "Coronavirus Disease 19" OR "Severe Acute Respiratory Syndrome Coronavirus 2 Infection" OR "SARS Coronavirus 2 Infection" OR "COVID-19 Virus Disease" OR "COVID 19 Virus Disease" OR "COVID-19 Virus Diseases" OR "Disease, COVID-19 Virus" OR "Virus Disease, COVID-19" OR "2019-nCoV Infection" OR "2019 nCoV Infection" OR "2019-nCoV Infections" OR "Infection, 2019-nCoV" OR "COVID19" OR "COVID-19 Pandemic" OR "COVID 19 Pandemic" OR "Pandemic, COVID-19" OR "COVID-19 Pandemics" OR "COVID-19 Testing"[Mesh] OR "COVID 19 Testing" OR "COVID-19 Testings" OR "Testing, COVID-19" OR "SARS Coronavirus 2 Testing" OR "COVID-19 Virus Testing" OR "COVID 19 Virus Testing" OR "COVID-19 Virus Testings" OR "Testing, COVID-19 Virus" OR "Virus Testing, COVID-19" OR "COVID19 Testing" OR "COVID19 Testings" OR "Testing, COVID19" OR "COVID19 Virus Testing" OR "COVID19 Virus Testings" OR "Testing, COVID19 Virus" OR "Virus Testing, COVID19" OR "SARS-CoV-2 Testing" OR "SARS CoV 2 Testing" OR "SARS-CoV-2 Testings" OR "Testing, SARS-CoV-2" OR "Coronavirus Disease 2019 Testing" OR "2019 Novel Coronavirus Disease Testing" OR "2019 Novel Coronavirus Testing" OR "2019-nCoV Disease Testing" OR "2019 nCoV Disease Testing" OR "2019-nCoV Disease Testings" OR "Disease Testing, 2019-nCoV" OR "Testing, 2019-nCoV Disease" OR "2019-nCoV Infection Testing" OR "2019 nCoV Infection Testing" OR "2019-nCoV Infection Testings" OR "Infection Testing, 2019-nCoV" OR "Testing, 2019-nCoV Infection" OR "COVID-19 Diagnostic Testing" OR "COVID 19 Diagnostic Testing" OR "COVID-19 Diagnostic Testings" OR "Diagnostic Testing, COVID-19" OR "Severe Acute Respiratory Syndrome Coronavirus 2 Testing" OR "Coronavirus Disease-19 Testing" OR "Coronavirus Disease 19 Testing" OR "Coronavirus Disease-19 Testings" OR "Testing, Coronavirus Disease-19" OR "2019-nCoV Testing" OR "2019 nCoV Testing" OR "2019-nCoV Testings" OR "Testing, 2019-nCoV" OR "COVID-19 Vaccines"[Mesh] OR "COVID 19 Vaccines" OR "Vaccines, COVID-19" OR "COVID-19 Virus Vaccines" OR "COVID 19 Virus Vaccines" OR "Vaccines, COVID-19 Virus" OR "Virus Vaccines, COVID-19" OR "COVID-19 Virus Vaccine" OR "COVID 19 Virus Vaccine" OR "Vaccine, COVID-19 Virus" OR "Virus Vaccine, COVID-19" OR "COVID19 Virus Vaccines" OR "Vaccines, COVID19 Virus" OR "Virus Vaccines, COVID19" OR "COVID19 Virus Vaccine" OR "Vaccine, COVID19 Virus" OR "Virus Vaccine, COVID19" OR "COVID19 Vaccines" OR "Vaccines, COVID19" OR "COVID19 Vaccine" OR "Vaccine, COVID19" OR "SARS-CoV-2 Vaccines" OR "SARS CoV 2 Vaccines" OR "Vaccines, SARS-CoV-2" OR "SARS-CoV-2 Vaccine" OR "SARS CoV 2 Vaccine" OR "Vaccine, SARS-CoV-2" OR "SARS2 Vaccines" OR "Vaccines, SARS2" OR "SARS2 Vaccine" OR "Vaccine, SARS2" OR "Coronavirus Disease 2019 Vaccines" OR "Coronavirus Disease 2019 Vaccine" OR "Coronavirus Disease 2019 Virus Vaccine" OR "Coronavirus Disease 2019 Virus Vaccines" OR "Coronavirus Disease-19 Vaccines" OR "Coronavirus Disease 19 Vaccines" OR "Vaccines, Coronavirus Disease-19" OR "Coronavirus Disease-19 Vaccine" OR "Coronavirus Disease 19 Vaccine" OR "Vaccine, Coronavirus Disease-19" OR "COVID 19 Vaccine" OR "Vaccine, COVID 19" OR "2019-nCoV Vaccine" OR "2019 nCoV Vaccine" OR "Vaccine, 2019-nCoV" OR "2019 Novel Coronavirus Vaccines" OR "2019 Novel Coronavirus Vaccine" OR "2019-nCoV Vaccines" OR "2019 nCoV Vaccines" OR "Vaccines, 2019-nCoV" OR "COVID-19 Vaccine" OR "Vaccine, COVID-19" OR "SARS Coronavirus 2 Vaccines"

Example to demonstrate the search for one disease (Dengue) by combining the three strings above:

("Afghanistan"[Mesh] OR “Afghanistan” OR "Bahrain"[Mesh] OR “Bahrain” OR "Djibouti"[Mesh] OR “Djibouti” OR “Somaliland, French” OR “Republic of Djibouti” OR “French Somaliland” OR "Egypt"[Mesh] OR "Arab Republic of Egypt" OR "United Arab Republic" OR "Iran"[Mesh] OR “Iran” OR “Islamic Republic of Iran” OR "Iraq"[Mesh] OR “Iraq” OR “Republic of Iraq” OR "Jordan"[Mesh] OR “Jordan” OR "Kuwait"[Mesh] OR “Kuwait” OR "Lebanon"[Mesh] OR “Lebanon” OR “Lebanese Republic” OR "Libya"[Mesh] OR “Libya” OR "Morocco"[Mesh] OR “Morocco” OR “Ifni” OR "Oman"[Mesh] OR “Oman” OR “Muscat” OR "Pakistan"[Mesh] OR “Pakistan” OR “Islamic Republic of Pakistan” OR “State of Palestine” OR “Occupied Palestinian Territory” OR "Qatar"[Mesh] OR “Qatar” OR “Katar” OR “State of Qatar” OR “Quatar” OR "Saudi Arabia"[Mesh] OR “Saudi Arabia” OR “Kingdom of Saudi Arabia” OR "Somalia"[Mesh] OR “Somalia” OR "Sudan"[Mesh] OR “Sudan” OR “Republic of the Sudan” OR "Syria"[Mesh] OR “Syria” OR “Syrian Arab Republic” OR "Tunisia"[Mesh] OR “Tunisia” OR "United Arab Emirates"[Mesh] OR “United Arab Emirates” OR “Trucial States” OR “Abu Dhabi” OR "Yemen"[Mesh] OR “Yemen” OR “Republic of Yemen” OR “Democratic Yemen” OR “Sanaa” OR “North Yemen” OR “Aden” OR “South Yemen”) AND ("Dengue"[Mesh] OR "Dengue" OR "Breakbone Fever" OR "Fever, Breakbone" OR "Classical Dengue Fever" OR "Classical Dengue Fevers" OR "Dengue Fever, Classical" OR "Break-Bone Fever" OR "Break Bone Fever" OR "Fever, Break-Bone" OR "Dengue Fever" OR "Fever, Dengue" OR "Classical Dengue" OR "Classical Dengues" OR "Dengue, Classical") AND (2011:2022[pdat])

S3: PubMed string used for studies on public health practices and interventions

EMR Countries AND (Broad Disease NOT Covid) AND Common Public Health Practices (MeSH Term is Public Health Practice) AND 2011-2022

("Afghanistan"[Mesh] OR "Bahrain"[Mesh] OR "Djibouti"[Mesh] OR "Egypt"[Mesh] OR "Iran"[Mesh] OR "Iraq"[Mesh] "Jordan"[Mesh] OR "Kuwait"[Mesh] "Lebanon"[Mesh] OR "Libya"[Mesh] OR "Morocco"[Mesh] OR "Oman"[Mesh] OR "Pakistan"[Mesh] OR "State of Palestine" OR "Occupied Palestinian Territory" OR "Qatar"[Mesh] OR "Saudi Arabia"[Mesh] OR "Somalia"[Mesh] OR "Sudan"[Mesh] OR "Syria"[Mesh] OR "Tunisia"[Mesh] OR "United Arab Emirates"[Mesh] OR "Yemen"[Mesh]) AND ("Zoonoses"[Mesh] OR "Vector Borne Diseases"[Mesh]) AND ("Public Health Practice/economics"[Mesh] OR "Public Health Practice/prevention and control"[Mesh]) Filters: from 2011 – 2022

S4: PubMed string used for socioeconomic impact of VBZDs

EMR Countries AND Broad Disease AND Socioeconomic Impact (MeSH Term is Socioeconomic Factors) AND 2011-2022

("Afghanistan"[Mesh] OR "Bahrain"[Mesh] OR "Djibouti"[Mesh] OR "Egypt"[Mesh] OR "Iran"[Mesh] OR "Iraq"[Mesh] "Jordan"[Mesh] OR "Kuwait"[Mesh] "Lebanon"[Mesh] OR "Libya"[Mesh] OR "Morocco"[Mesh] OR "Oman"[Mesh] OR "Pakistan"[Mesh] OR "State of Palestine" OR "Occupied Palestinian Territory" OR "Qatar"[Mesh] OR "Saudi Arabia"[Mesh] OR "Somalia"[Mesh] OR "Sudan"[Mesh] OR "Syria"[Mesh] OR "Tunisia"[Mesh] OR "United Arab Emirates"[Mesh] OR "Yemen"[Mesh]) AND ("Zoonoses"[Mesh] OR "Vector Borne Diseases"[Mesh]) AND ("Socioeconomic Factors"[Mesh]) Filters: from 2011 – 2022

S5: Variables extracted

year of publication
disease
year of study
country
study types
study setting
data level
age group
sex
data source
outbreak investigation
incidence age difference
incidence sex difference
incidence-age comparison
incidence geography difference
mortality sex
mortality age
mortality geography
socioeconomic differences
incidence time trend
mortality time trend
treatment availability quality
testing availability quality
vector burden
overall disease control
overall vector control
overall economic impact
overall pub health measures

S6: Overview of studies included in this analysis

Disease	Number of studies[#] (N= 295)	Number of countries (N=17)	Major countries (% of studies)	Years of publication (First-last year)
CCHF	7 (2.4%)	5	Iran (43%)	2008-2020
Chikungunya	19 (6.4%)	7*	Pakistan (47%)	2014-2022
DF	77 (26%)	7	Pakistan (55%)	2011-2022
Zoonotic Influenza A	41 (14%)	9	Iran (32%)	2011-2022
Leishmaniasis	87 (29%)	11	Iran (48%)	2011-2022
Leptospirosis	6 (2.0%)	2	Iran (83%)	2011-2020
MERS	35 (12%)	3	Saudi Arabia (91%)	2014-2021
Rabies	7 (2.4%)	5	Pakistan and Tunisia (29% each)	2012-2021
RVF	11 (3.7%)	4	Saudi Arabia (45%)	2011-2020
WNF	4 (1.4%)	3	Tunisia (50%)	2012-2017
YF	1 (0.3%)	1	Sudan (100%)	2013

Notes:

# percentage denotes the proportion of total studies

**One study on Chikungunya had data across Iran and Pakistan border.*

CCHF— Crimean-Congo hemorrhagic fever; DF—Dengue Fever; MERS— Middle East Respiratory Syndrome; RVF—Rift Valley Fever; WNF—West Nile Fever; YF—Yellow Fever

S7: Summary of key findings from studies- disease wise

Diseases/ number of studies	CCHF *N=7*	CHIK *N=19*	DENG *N=77*	ZINF *N=41*	LEISH *N=87*	LEP *N=6*	MERS *N=35*	RAB *N=7*	RVF *N=11*	WNF *N=4*	YF *N=1*
Year of publication											
2011	0 (0%)	0 (0%)	4 (5.2%)	5 (12%)	4 (4.6%)	1 (17%)	0 (0%)	0 (0%)	1 (9.1%)	0 (0%)	0 (0%)
2012	1 (14%)	0 (0%)	7 (9.1%)	6 (15%)	6 (6.9%)	1 (17%)	0 (0%)	1 (14%)	1 (9.1%)	1 (25%)	0 (0%)
2013	0 (0%)	0 (0%)	5 (6.5%)	2 (4.9%)	10 (11%)	0 (0%)	0 (0%)	0 (0%)	1 (9.1%)	0 (0%)	1 (100%)
2014	0 (0%)	2 (11%)	10 (13%)	4 (9.8%)	9 (10%)	1 (17%)	3 (8.6%)	0 (0%)	1 (9.1%)	1 (25%)	0 (0%)
2015	0 (0%)	0 (0%)	2 (2.6%)	3 (7.3%)	11 (13%)	0 (0%)	4 (11%)	1 (14%)	2 (18%)	0 (0%)	0 (0%)
2016	0 (0%)	0 (0%)	4 (5.2%)	2 (4.9%)	17 (20%)	0 (0%)	7 (20%)	0 (0%)	1 (9.1%)	0 (0%)	0 (0%)
2017	1 (14%)	0 (0%)	11 (14%)	1 (2.4%)	5 (5.7%)	1 (17%)	5 (14%)	0 (0%)	0 (0%)	2 (50%)	0 (0%)
2018	1 (14%)	2 (11%)	8 (10%)	3 (7.3%)	6 (6.9%)	0 (0%)	2 (5.7%)	0 (0%)	0 (0%)	0 (0%)	0 (0%)
2019	2 (29%)	3 (16%)	7 (9.1%)	3 (7.3%)	9 (10%)	1 (17%)	5 (14%)	1 (14%)	3 (27%)	0 (0%)	0 (0%)
2020	1 (14%)	5 (26%)	12 (16%)	3 (7.3%)	2 (2.3%)	1 (17%)	5 (14%)	0 (0%)	1 (9.1%)	0 (0%)	0 (0%)
2021	1 (14%)	6 (32%)	4 (5.2%)	5 (12%)	4 (4.6%)	0 (0%)	4 (11%)	4 (57%)	0 (0%)	0 (0%)	0 (0%)
2022	0 (0%)	1 (5.3%)	3 (3.9%)	4 (9.8%)	4 (4.6%)	0 (0%)	0 (0%)	0 (0%)	0 (0%)	0 (0%)	0 (0%)
Country											
Afghanistan	1 (14%)	0 (0%)	0 (0%)	0 (0%)	0 (0%)	0 (0%)	0 (0%)	0 (0%)	0 (0%)	0 (0%)	0 (0%)
Djibouti	0 (0%)	1 (5.3%)	0 (0%)	0 (0%)	0 (0%)	0 (0%)	0 (0%)	0 (0%)	1 (9.1%)	0 (0%)	0 (0%)
Egypt	0 (0%)	0 (0%)	2 (2.6%)	4 (9.8%)	0 (0%)	0 (0%)	0 (0%)	0 (0%)	1 (9.1%)	0 (0%)	0 (0%)
Iran	3 (43%)	2 (11.0%)	0 (0%)	13 (32%)	42 (48%)	5 (83%)	0 (0%)	1 (14%)	0 (0%)	1 (25%)	0 (0%)
Iraq	0 (0%)	0 (0%)	0 (0%)	0 (0%)	1 (1.1%)	0 (0%)	0 (0%)	0 (0%)	0 (0%)	0 (0%)	0 (0%)
Jordan	0 (0%)	0 (0%)	0 (0%)	0 (0%)	1 (1.1%)	0 (0%)	0 (0%)	0 (0%)	0 (0%)	0 (0%)	0 (0%)
Libya	0 (0%)	0 (0%)	0 (0%)	0 (0%)	1 (1.1%)	0 (0%)	0 (0%)	0 (0%)	0 (0%)	0 (0%)	0 (0%)
Morocco	0 (0%)	0 (0%)	0 (0%)	1 (2.4%)	7 (8.0%)	1 (17%)	0 (0%)	0 (0%)	0 (0%)	0 (0%)	0 (0%)
Oman	1 (14%)	0 (0%)	2 (2.6%)	0 (0%)	0 (0%)	0 (0%)	0 (0%)	1 (14%)	0 (0%)	0 (0%)	0 (0%)
Pakistan	1 (14%)	9 (47%)	42 (55%)	9 (22%)	11 (13%)	0 (0%)	0 (0%)	2 (29%)	0 (0%)	1 (25%)	0 (0%)
Qatar	0 (0%)	1 (5.3%)	1 (1.3%)	1 (2.4%)	0 (0%)	0 (0%)	2 (5.7%)	0 (0%)	0 (0%)	0 (0%)	0 (0%)
KSA	0 (0%)	2 (11%)	16 (21%)	7 (17%)	6 (6.9%)	0 (0%)	32 (91%)	0 (0%)	5 (45%)	0 (0%)	0 (0%)
Sudan	1 (14%)	3 (16%)	12 (16%)	0 (0%)	9 (10%)	0 (0%)	0 (0%)	0 (0%)	4 (36%)	0 (0%)	1 (100%)
Syria	0 (0%)	0 (0%)	0 (0%)	0 (0%)	1 (1.1%)	0 (0%)	0 (0%)	0 (0%)	0 (0%)	0 (0%)	0 (0%)
Tunisia	0 (0%)	0 (0%)	0 (0%)	3 (7.3%)	2 (2.3%)	0 (0%)	0 (0%)	2 (29%)	0 (0%)	2 (50%)	0 (0%)
UAE	0 (0%)	0 (0%)	0 (0%)	2 (4.9%)	0 (0%)	0 (0%)	1 (2.9%)	0 (0%)	0 (0%)	0 (0%)	0 (0%)
Yemen	0 (0%)	1 (5.3%)	2 (2.6%)	1 (2.4%)	6 (6.9%)	0 (0%)	0 (0%)	1 (14%)	0 (0%)	0 (0%)	0 (0%)
Study designs											
case control	0 (0%)	0 (0%)	5 (6.5%)	0 (0%)	4 (4.6%)	1 (17%)	2 (5.7%)	0 (0%)	0 (0%)	0 (0%)	0 (0%)

Diseases/ number of studies	CCHF *N=7*	CHIK *N=19*	DENG *N=77*	ZINF *N=41*	LEISH *N=87*	LEP *N=6*	MERS *N=35*	RAB *N=7*	RVF *N=11*	WNF *N=4*	YF *N=1*
cohort	0 (0%)	1 (5.3%)	2 (2.6%)	11 (27%)	3 (3.4%)	0 (0%)	5 (14%)	0 (0%)	0 (0%)	0 (0%)	0 (0%)
cross sectional	4 (57%)	11 (58%)	37 (48%)	8 (20%)	46 (53%)	1 (17%)	19 (54%)	0 (0%)	2 (18%)	0 (0%)	1 (100%)
interventional	0 (0%)	0 (0%)	0 (0%)	0 (0%)	1 (1.1%)	0 (0%)	0 (0%)	0 (0%)	0 (0%)	0 (0%)	0 (0%)
longitudinal	0 (0%)	0 (0%)	0 (0%)	0 (0%)	0 (0%)	0 (0%)	0 (0%)	2 (29%)	0 (0%)	0 (0%)	0 (0%)
multi-year C.S	3 (43%)	6 (32%)	27 (35%)	22 (54%)	29 (33%)	4 (67%)	6 (17%)	3 (43%)	6 (55%)	4 (100%)	0 (0%)
other designs	0 (0%)	1 (5.3%)	6 (7.8%)	0 (0%)	4 (4.6%)	0 (0%)	3 (8.6%)	2 (29%)	3 (27%)	0 (0%)	0 (0%)
**Study setting**											
community	6 (86%)	5 (26%)	19 (25%)	11 (27%)	37 (43%)	1 (17%)	1 (2.9%)	2 (29%)	6 (55%)	0 (0%)	1 (100%)
hospital based	1 (14%)	13 (68%)	55 (71%)	27 (66%)	47 (54%)	3 (50%)	26 (74%)	5 (71%)	5 (45%)	4 (100%)	0 (0%)
mixed	0 (0%)	1 (5.3%)	3 (3.9%)	3 (7.3%)	3 (3.4%)	2 (33%)	8 (23%)	0 (0%)	0 (0%)	0 (0%)	0 (0%)
**Data level**											
national	1 (14%)	0 (0%)	5 (6.5%)	8 (20%)	3 (3.4%)	0 (0%)	8 (23%)	3 (43%)	0 (0%)	0 (0%)	0 (0%)
subnational	6 (86%)	19 (100%)	72 (94%)	33 (80%)	84 (97%)	6 (100%)	27 (77%)	4 (57%)	11 (100%)	4 (100%)	1 (100%)
**Age group**											
all adults	3 (43%)	1 (5.3%)	6 (7.8%)	0 (0%)	0 (0%)	0 (0%)	1 (2.9%)	0 (0%)	2 (18%)	2 (50%)	0 (0%)
all age groups	2 (29%)	9 (47%)	58 (75%)	25 (61%)	68 (78%)	4 (67%)	32 (91%)	7 (100%)	4 (36%)	2 (50%)	0 (0%)
children/<18Y	0 (0%)	1 (5.3%)	1 (1.3%)	4 (9.8%)	6 (6.9%)	0 (0%)	0 (0%)	0 (0%)	1 (9.1%)	0 (0%)	0 (0%)
non-elderly adults	2 (29%)	2 (11%)	3 (3.9%)	8 (20%)	4 (4.6%)	0 (0%)	2 (5.7%)	0 (0%)	0 (0%)	0 (0%)	1 (100%)
NA	0 (0%)	6 (32%)	9 (12%)	4 (9.8%)	9 (10%)	2 (33%)	0 (0%)	0 (0%)	4 (36%)	0 (0%)	0 (0%)
**Sex**											
both male and female	3 (43%)	13 (68%)	68 (88%)	32 (78%)	72 (83%)	4 (67%)	35 (100%)	7 (100%)	6 (55%)	4 (100%)	1 (100%)
female	1 (14%)	1 (5.3%)	1 (1.3%)	1 (2.4%)	1 (1.1%)	0 (0%)	0 (0%)	0 (0%)	1 (9.1%)	0 (0%)	0 (0%)
male	3 (43%)	1 (5.3%)	3 (3.9%)	3 (7.3%)	2 (2.3%)	0 (0%)	0 (0%)	0 (0%)	0 (0%)	0 (0%)	0 (0%)
NA	0 (0%)	4 (21%)	5 (6.5%)	5 (12%)	12 (14%)	2 (33%)	0 (0%)	0 (0%)	4 (36%)	0 (0%)	0 (0%)
**Data source**											
both	0 (0%)	1 (5.3%)	4 (5.2%)	1 (2.4%)	1 (1.1%)	0 (0%)	6 (17%)	0 (0%)	1 (9.1%)	1 (25%)	0 (0%)
NA	0 (0%)	0 (0%)	1 (1.3%)	0 (0%)	0 (0%)	1 (17%)	0 (0%)	0 (0%)	0 (0%)	0 (0%)	0 (0%)
primary data	6 (86%)	18 (95%)	62 (81%)	40 (98%)	72 (83%)	5 (83%)	8 (23%)	3 (43%)	9 (82%)	3 (75%)	1 (100%)
secondary data	1 (14%)	0 (0%)	10 (13%)	0 (0%)	14 (16%)	0 (0%)	21 (60%)	4 (57%)	1 (9.1%)	0 (0%)	0 (0%)
**Outbreak**	2 (29%)	1 (5.3%)	15 (19%)	16 (39%)	11 (13%)	0 (0%)	5 (14%)	0 (0%)	1 (9.1%)	0 (0%)	1 (100%)

Note: [1] n (%); CCHF— Crimean-Congo hemorrhagic fever; CHIK— Chikungunya; DENG—Dengue Fever; MERS— Middle East Respiratory Syndrome; ZINF—Zoonotic influenzas; LEISH— Leishmaniasis; LEP—Leptospirosis; RAB—Rabies; RVF—Rift Valley Fever; WNF—West Nile Fever; YF—Yellow Fever; NA— Not available/applicable; C.S— cross-sectional study

S8: List of studies included in the analysis of seroprevalence rates for VBZDs in EMR

Disease	Year	Country	Data level	Age groups	Sex	Indicator	Sample	Cases	Values
CL	2014	Iran	subnational	all age groups	both	incidence	904	124	13.70%
CL	2015	Yemen	subnational	all age groups	both	seroprevalence	525	98.7	18.80%
CL	2019	Pakistan	subnational	all age groups	both	seroprevalence	1674	1674	50.40%
CL	##	Pakistan	subnational	all age groups	both	PCR positivity	339	265	78.20%
CL	##	Iran	subnational	all age groups	both	Prevalence[1]	5098	326	6.40%
CL	##	Yemen	subnational	all age groups	both	Seroprevalence[2]	1165	216	18.50%
CL	##	Pakistan	subnational	all age groups	both	seroprevalence	300	172	57.30%
CL	##	Iran	subnational	all age groups	both	seroprevalence	5544	67	1.20%
CCHF	2014	Iran	subnational	non-elderly adults	male	seroprevalence	136	39	28.68%
CCHF	2020	Iran	subnational	all adults	male	seroprevalence	100	7	7.00%
CCHF	##	Iran	subnational	all adults	male	seroprevalence	190	31	16.50%
CCHF	##	Pakistan	subnational	all adults	female	seroprevalence	453	26	5.74%
CHIK	2018	Iran	subnational	NA	both	seroprevalence	159	40	25.20%
CHIK	2020	Djibouti	subnational	all age groups	both	seroprevalence	911	24	2.60%
CHIK	##	KSA	subnational	NA	NA	seroprevalence	40	1	2.50%
DEN	2010	Sudan	subnational	NA	NA	incidence	3765	35	0.94%
DEN	2011	KSA	national	all age groups	both	seroprevalence	965	306	31.70%
DEN	2011	Sudan	subnational	all age groups	both	seroprevalence	489	49	9.40%
DEN	2012	Sudan	subnational	all adults	both	seroprevalence	600	166	27.70%
DEN	2016	Pakistan	subnational	children/<18 years	both	seroprevalence	400	100	25.00%
DEN	2018	Djibouti	subnational	all age groups	both	seroprevalence	1045	20	21.80%
DEN	2018	Oman	subnational	NA	both	seroprevalence	343	59	17.20%
DEN	2019	Egypt	subnational	all age groups	both	seroprevalence	91	11	12.09%
DEN	##	Sudan	subnational	all adults	both	seroprevalence	701	80	11.42%
DEN	##	Sudan	subnational	all age groups	both	incidence	54886	193	0.35%
DEN	##	Sudan	subnational	all age groups	both	seroprevalence	701	334	47.60%
DEN	##	Sudan	subnational	all age groups	both	seroprevalence	791	41	5.18%
DEN	##	Sudan	subnational	all age groups	both	seroprevalence	448	302	77.00%
DEN	##	KSA	national	all age groups	both	seroprevalence	6596	1710	26.70%
DEN	##	Sudan	subnational	NA	NA	seroprevalence	157	66	42.04%
H1N1	##	Egypt	subnational	all age groups	both	incidence	2383	95	4.00%
H1N1	##	Pakistan	national	all age groups	both	seroprevalence	1243	261	21.00%

Disease	Year	Country	Data level	Age groups	Sex	Indicator	Sample	Cases	Values
H1N1/H3N3	##	Iran	subnational	all age groups	both	PCR positivity	1269	191	15.10%
H5N1	##	Egypt	subnational	all age groups	both	seroprevalence	750	56.25	7.50%
H9N2	2010	Iran	subnational	non-elderly adults	both	seroprevalence	100	3	3.00%
H9N2	##	Pakistan	subnational	non-elderly adults	male	seroprevalence	332	167	50.30%
H9N2	##	Iran	subnational	NA	NA	seroprevalence	182	21	11.50%
LEPTO	##	Iran	subnational	all age groups	both	seroprevalence	250	50	20%
MERS	##	UAE	subnational	non-elderly adults	both	seroprevalence	235	40	17.00%
RVF	2018	Djibouti	subnational	all age groups	both	seroprevalence	1045	20	2.20%
RVF	2019	KSA	subnational	NA	NA	seroprevalence	80	0	0%
RVF	##	KSA	subnational	all adults	both	seroprevalence	350	39	11.14%
VL	2011	Iran	subnational	non-elderly adults	NA	seroprevalence	1800	19	0.33%
VL	2012	Iran	subnational	all age groups	NA	seroprevalence	2003	28	1.40%
VL	2013	Iran	subnational	all age groups	both	seroprevalence	1476	22	1.50%
VL	2013	Iran	subnational	NA	NA	seroprevalence	456	21	0.43%
VL	2014	Iran	subnational	children/<18 years	NA	seroprevalence	1007	37	3.70%
VL	##	Iran	subnational	all age groups	NA	seroprevalence	9396	403	4.30%
WNF	2020	Djibouti	subnational	all age groups	both	seroprevalence	911	6	0.60%
YF	2017	Sudan	subnational	non-elderly adults	both	seroprevalence	16	4	25.00%
CL	##	Morocco	subnational	NA	NA	Incidence rate	-	-	4.1/1,000 persons/year
VL	##	Sudan	subnational	all age groups	both	Incidence rate	-	-	7.0/1000 persons/year

Note: [1]— prevalence measured using microscopic exam of parasite from ulcer; [2]— prevalence measured using Leishmanin Skin Test; CL— Cutaneous Leishmaniasis; VL— Visceral Leishmaniasis; RVF— Rift Valley Fever; WNF— West Nile Fever; YF— Yellow Fever; LEPTO—Leptospirosis; DEN— Dengue Fever; CHIK- Chikungunya; ##- multi-year data; NA— Not applicable/not available; KSA— Kingdom of Saudi Arabia

S9: List of studies included in the review

First author; year	Disease	Country	Study types	Study setting	Data level	Age group	Sex
Al-Abri SS et al;2019	CCHF	Oman	CS	HB	national	non-elderly adults	both
Bower, H. et al;2019	CCHF	Sudan	CS	CB	subnational	all age groups	both
Mofleh, J. et al;2012	CCHF	Afghanistan	MY C/S	CB	subnational	all age groups	both
Mostafavi, E. et al;2017	CCHF	Iran	MY C/S	CB	subnational	all adults	male
Salmanzadeh, S. et al;2021	CCHF	Iran	CS	CB	subnational	all adults	male
Shahhosseini, N. et al;2018	CCHF	Iran	CS	CB	subnational	non-elderly adults	male
Shahid, M.F. et al;2020	CCHF	Pakistan	MY C/S	CB	subnational	all adults	both
Ali, A.A.A. et al;2022	CHIK	Sudan	CS	HB	subnational	non-elderly adults	female
Andayi, F. et al;2014	CHIK	Djibouti	CS	CB	subnational	all age groups	both
Badar N et al;2020	CHIK	Pakistan	MY C/S	HB	subnational	all age groups	both
Badar N et al;2021	CHIK	Pakistan	CS	HB	subnational	all age groups	both
Badar N et al;2021	CHIK	Pakistan	MY C/S	HB	subnational	non-elderly adults	both
Bower, H. et al;2021	CHIK	Sudan	C	HB	subnational	all age groups	both
Hakami, A.R. et al;2021	CHIK	KSA	MY C/S	CB	subnational	NA/NS	NA/NS
Hira FS et al;2018	CHIK	Pakistan	CS	CB	subnational	NA/NS	NA/NS
Humphrey, J.M. et al;2019	CHIK	Qatar	CS	HB	subnational	all adults	male
Kam, Y.-W. et al;2020	CHIK	KSA	CS	HB	subnational	NA/NS	both
Malik, M.R. et al;2014	CHIK	Yemen	MY C/S	HB	subnational	all age groups	both
Meraj L et al;2020	CHIK	Pakistan	MY C/S	HB	subnational	children/<18	both
Mohamed N et al;2019	CHIK	Sudan	CS	HB	subnational	all age groups	both
Nejati, J. et al;2020	CHIK	Iran	CS	CB	subnational	all age groups	NA/NS
Pouriayevali MH et al;2019	CHIK	Iran	CS	CB	subnational	NA/NS	both
Prakoso D et al;2021	CHIK	Pakistan	MY C/S	HB	subnational	NA/NS	NA/NS
Raza FA et al;2021	CHIK	Pakistan	CS	Mx	subnational	all age groups	both
Shahid U et al;2020	CHIK	Pakistan	Other	HB	subnational	all age groups	both
Zaman, H. et al;2018	CHIK	Pakistan	CS	HB	subnational	NA/NS	both

First author; year	Disease	Country	Study types	Study setting	Data level	Age group	Sex
Abualamah WA et al;2020	DF	KSA	CC	HB	subnational	all age groups	both
Adam, A. et al;2018	DF	Sudan	MY C/S	CB	subnational	all age groups	both
Ahmad Qureshi EM et al;2017	DF	Pakistan	Other	CB	subnational	all age groups	both
Ahmed, A. et al;2019	DF	Sudan	CS	HB	subnational	all age groups	both
Ahmed, A. et al;2019	DF	Sudan	MY C/S	HB	subnational	all age groups	both
Ahmed, S. et al;2013	DF	Pakistan	CS	HB	subnational	all age groups	both
Akbar, N.A. et al;2020	DF	KSA	MY C/S	HB	subnational	all age groups	both
Al Awaidy ST et al;2014	DF	Oman	C	HB	national	all age groups	both
Al-Abri, S.S. et al;2020	DF	Oman	CS	CB	subnational	NA/NS	both
Al-Azraqi TA et al;2013	DF	KSA	CS	CB	national	all age groups	both
Al-Raddadi R et al;2019	DF	KSA	MY C/S	CB	national	all age groups	both
Alghazali, K.A. et al;2020	DF	Yemen	CS	HB	subnational	all age groups	both
Ali A et al;2013	DF	Pakistan	CS	HB	subnational	all age groups	both
Anwar, F. et al;2020	DF	Pakistan	CS	HB	subnational	all age groups	both
Ashshi AM;2017	DF	KSA	MY C/S	HB	subnational	non-elderly adults	male
Ashshi, A.M. et al;2017	DF	KSA	MY C/S	HB	subnational	non-elderly adults	male
Ashshi, A.M.;2015	DF	KSA	CS	HB	subnational	non-elderly adults	male
Assir MZ et al;2014	DF	Pakistan	CS	HB	subnational	all age groups	both
Assir, M.Z.K. et al;2014	DF	Pakistan	CS	HB	subnational	all age groups	both
Atique S et al;2018	DF	Pakistan	Other	HB	subnational	all age groups	both
Attaullah, et al;2017	DF	Pakistan	MY C/S	HB	subnational	all age groups	both
Awan NJ et al;2022	DF	Pakistan	CC	HB	subnational	all age groups	both
Aziz AT et al;2014	DF	KSA	CS	CB	subnational	all age groups	both
Badreddine S et al;2017	DF	KSA	MY C/S	HB	subnational	all age groups	both
Badshah, A. et al;2019	DF	Pakistan	CS	HB	subnational	all age groups	both
Bin Ghouth AS et al;2012	DF	Yemen	Other	HB	subnational	all age groups	both
Chaudhry, M. et al;2017	DF	Pakistan	CS	HB	subnational	all age groups	both
El-Kady, A.M. et al;2022	DF	Egypt	CS	HB	subnational	all age groups	both

First author; year	Disease	Country	Study types	Study setting	Data level	Age group	Sex
Elaagip A et al;2020	DF	Sudan	MY C/S	CB	subnational	NA/NS	NA/NS
Eldigail MH et al;2018	DF	Sudan	MY C/S	CB	subnational	all age groups	both
Eldigail MH et al;2020	DF	Sudan	MY C/S	CB	subnational	all adults	both
Gamil, M.A. et al;2014	DF	KSA	MY C/S	HB	subnational	all age groups	both
Ghani E et al;2017	DF	Pakistan	CS	HB	subnational	all age groups	both
Hashem AM et al;2018	DF	KSA	C	HB	subnational	all age groups	both
Hegazi MA et al;2020	DF	KSA	MY C/S	HB	subnational	all age groups	both
Humphrey, J.M. et al;2019	DF	Qatar	MY C/S	HB	subnational	all adults	both
Hussen, M.O. et al;2020	DF	Egypt	CS	CB	subnational	all age groups	both
Iqtadar, S. et al;2017	DF	Pakistan	CS	HB	subnational	all age groups	both
Jamjoom, G.A. et al;2016	DF	KSA	MY C/S	HB	subnational	all age groups	both
Khadija Rafique, Sheikh et al;2012	DF	Pakistan	CS	HB	subnational	all age groups	both
Khalil MA et al;2014	DF	Pakistan	MY C/S	HB	subnational	all age groups	both
Khan J et al;2018	DF	Pakistan	MY C/S	Mx	subnational	all age groups	both
Khan NU et al;2020	DF	Pakistan	CS	HB	subnational	all age groups	both
Khan, J. et al;2016	DF	Pakistan	CS	CB	subnational	all age groups	both
Kholedi AA et al;2012	DF	KSA	CC	HB	subnational	all age groups	both
Khormi HM et al;2011	DF	KSA	MY C/S	Mx	subnational	all age groups	both
Kiran, Ejaz et al;2011	DF	Pakistan	Other	HB	subnational	NA/NS	both
Malik, A. et al;2011	DF	Sudan	MY C/S	HB	subnational	all age groups	both
Malik, A. et al;2017	DF	Pakistan	MY C/S	CB	subnational	NA/NS	NA/NS
Mehmood A et al;2021	DF	Pakistan	CC	CB	subnational	all age groups	both
Melebari S et al;2021	DF	KSA	MY C/S	HB	subnational	all age groups	both
Mohamud MA et al;2020	DF	Pakistan	CS	HB	subnational	all age groups	both
Mohsin SN et al;2016	DF	Pakistan	CS	CB	subnational	children/<18	both
Mubashir, M. et al;2020	DF	Pakistan	MY C/S	HB	subnational	all adults	female
Muhammad, I. et al;2016	DF	Pakistan	CS	HB	subnational	all age groups	both
Mukhtar F et al;2012	DF	Pakistan	CS	HB	subnational	all age groups	both

First author; year	Disease	Country	Study types	Study setting	Data level	Age group	Sex
Mukhtar MU et al;2018	DF	Pakistan	CS	HB	subnational	all age groups	both
Mukhtar MU et al;2021	DF	Pakistan	MY C/S	HB	subnational	all age groups	both
Murad H et al;2014	DF	Pakistan	CC	HB	subnational	all age groups	both
Noureldin E et al;2019	DF	Sudan	Other	CB	subnational	NA/NS	both
Rana, M.S. et al;2012	DF	Pakistan	CS	HB	subnational	all age groups	NA/NS
Rauf A et al;2017	DF	Pakistan	CS	HB	subnational	all adults	both
Raza FA et al;2018	DF	Pakistan	CS	HB	subnational	all age groups	both
Raza FA et al;2021	DF	Pakistan	MY C/S	Mx	national	all age groups	both
Raza, F.A. et al;2014	DF	Pakistan	MY C/S	HB	subnational	all age groups	both
Rehman, A.U. et al;2022	DF	Pakistan	CS	HB	subnational	all age groups	both
Saba, S. et al;2019	DF	Pakistan	CS	HB	subnational	all age groups	both
Saqib MA et al;2014	DF	Pakistan	CS	HB	subnational	all age groups	both
Sayed, Himatt et al;2015	DF	Sudan	CS	CB	subnational	all age groups	both
Seidahmed OM et al;2012	DF	Sudan	Other	CB	subnational	all age groups	both
Seidahmed, O.M.E. et al;2012	DF	Sudan	CS	CB	subnational	NA/NS	NA/NS
Soghaier, M.A. et al;2014	DF	Sudan	CS	CB	subnational	all adults	both
Suleman M et al;2017	DF	Pakistan	MY C/S	HB	national	all age groups	both
Syed Riazul, Hasan et al;2013	DF	Pakistan	CS	HB	subnational	all age groups	both
Yousaf, M. et al;2018	DF	Pakistan	MY C/S	HB	subnational	all age groups	both
Zafar H et al;2011	DF	Pakistan	CS	HB	subnational	NA/NS	NA/NS
Zafar H et al;2013	DF	Pakistan	CS	HB	subnational	all adults	both
Abdalla, O. et al;2020	ZIA	KSA	MY C/S	HB	national	all age groups	both
Ahad A et al;2014	ZIA	Pakistan	MY C/S	CB	subnational	non-elderly adults	male
Ahmadreza, Moradi et al;2011	ZIA	Iran	MY C/S	HB	subnational	all age groups	both
Al Amad M et al;2022	ZIA	Yemen	C	HB	national	all age groups	both
Al Sheikh, R.H. et al;2015	ZIA	KSA	CS	CB	national	NA/NS	NA/NS
Al Subaie SS et al;2012	ZIA	KSA	C	HB	subnational	children/<18	both
Al-Romaihi, H.E. et al;2020	ZIA	Qatar	MY C/S	HB	national	children/<18	both

First author; year	Disease	Country	Study types	Study setting	Data level	Age group	Sex
Al-Tawfiq JA et al;2011	ZIA	KSA	C	HB	subnational	all age groups	both
Alavi, S.M. et al;2012	ZIA	Iran	MY C/S	HB	subnational	children/<18	both
Alavi, S.M. et al;2014	ZIA	Iran	MY C/S	HB	subnational	all age groups	both
Ali SA et al;2013	ZIA	Pakistan	C	HB	subnational	children/<18	male
Altayep, K.M. et al;2017	ZIA	KSA	MY C/S	HB	subnational	non-elderly adults	both
Anvar, E. et al;2013	ZIA	Iran	CS	CB	subnational	NA/NS	NA/NS
Behnaz, F. et al;2012	ZIA	Iran	CS	CB	subnational	all age groups	both
Bouneb R et al;2018	ZIA	Tunisia	MY C/S	HB	subnational	non-elderly adults	both
Chaudhry M et al;2020	ZIA	Pakistan	MY C/S	Mx	subnational	non-elderly adults	both
Damak H et al;2011	ZIA	Tunisia	C	HB	subnational	all age groups	both
F., Ahmed et al;2012	ZIA	UAE	C	HB	national	all age groups	both
Faezi, N.A. et al;2016	ZIA	Iran	MY C/S	HB	subnational	all age groups	both
Fahim, M. et al;2021	ZIA	Egypt	MY C/S	HB	national	all age groups	both
Faramarzi, H. et al;2021	ZIA	Iran	MY C/S	CB	subnational	all age groups	both
Gomaa, M.R. et al;2015	ZIA	Egypt	C	CB	subnational	all age groups	both
Gomaa, M.R. et al;2022	ZIA	Egypt	C	CB	subnational	all age groups	both
Haghshenas, M. et al;2015	ZIA	Iran	CS	HB	subnational	all age groups	both
Hasan, S. et al;2021	ZIA	Pakistan	CS	HB	subnational	all age groups	both
Hasan, S. et al;2022	ZIA	Pakistan	MY C/S	HB	subnational	all age groups	both
Hashemi, S.A. et al;2021	ZIA	Iran	MY C/S	HB	subnational	all age groups	both
Heidari, A. et al;2016	ZIA	Iran	CS	CB	subnational	non-elderly adults	both
Jamoussi A et al;2022	ZIA	Tunisia	C	HB	subnational	non-elderly adults	both
Khan G et al;2011	ZIA	UAE	MY C/S	HB	subnational	all age groups	both
Khanum, I. et al;2021	ZIA	Pakistan	C	HB	subnational	non-elderly adults	NA/NS
Lahlou Amine I et al;2011	ZIA	Morocco	C	HB	subnational	all age groups	both
Mady A et al;2012	ZIA	KSA	MY C/S	HB	subnational	all age groups	both
Mood, F.S. et al;2019	ZIA	Iran	CS	HB	subnational	all age groups	both
Nisar N et al;2014	ZIA	Pakistan	MY C/S	CB	national	all age groups	both

First author; year	Disease	Country	Study types	Study setting	Data level	Age group	Sex
Noor, M. et al;2019	ZIA	Pakistan	MY C/S	HB	subnational	all age groups	both
Rabaan AA et al;2018	ZIA	KSA	CS	HB	subnational	all age groups	both
Sheta, B.M. et al;2014	ZIA	Egypt	MY C/S	CB	subnational	NA/NS	NA/NS
Tahir MF et al;2019	ZIA	Pakistan	MY C/S	CB	subnational	non-elderly adults	male
Yavarian, J. et al;2018	ZIA	Iran	MY C/S	Mx	national	all age groups	both
Zarinfar, N. et al;2012	ZIA	Iran	MY C/S	Mx	subnational	all age groups	both
Aasri, A.E. et al;2016	LEISH	Morocco	CS	HB	subnational	all age groups	both
Abdellatif MZ et al;2013	LEISH	Libya	CS	HB	subnational	NA/NS	both
Abdi, J. et al;2015	LEISH	Iran	CS	CB	subnational	all age groups	NA/NS
Abedi-Astaneh, F. et al;2016	LEISH	Iran	Other	CB	subnational	all age groups	both
Abolghazi, A. et al;2019	LEISH	Iran	MY C/S	HB	subnational	all age groups	both
Adam GK et al;2016	LEISH	Sudan	MY C/S	HB	national	all age groups	both
Adam GK et al;2018	LEISH	Sudan	CS	HB	subnational	non-elderly adults	female
Aflatoonian, M.R. et al;2013	LEISH	Iran	MY C/S	CB	subnational	all age groups	both
Aflatoonian, M.R. et al;2014	LEISH	Iran	C	CB	subnational	all age groups	both
Ahmad, S. et al;2022	LEISH	Pakistan	CS	CB	subnational	all age groups	both
Ahmadi, N.A. et al;2013	LEISH	Iran	CS	HB	subnational	all age groups	both
Ahmed MA et al;2016	LEISH	Sudan	MY C/S	HB	subnational	children/<18	both
Ahmed, M.A.A. et al;2022	LEISH	Sudan	MY C/S	HB	subnational	all age groups	both
Akhoundi, M. et al;2013	LEISH	Iran	CS	HB	subnational	all age groups	both
Al-Kamel MA;2016	LEISH	Yemen	MY C/S	HB	subnational	all age groups	both
Alanazi, A.D. et al;2016	LEISH	KSA	CS	HB	subnational	all age groups	both
Ali Nilforoushzadeh, M. et al;2014	LEISH	Iran	MY C/S	HB	subnational	non-elderly adults	both
Alkulaibi, M.M. et al;2019	LEISH	Yemen	MY C/S	CB	subnational	all age groups	both
Alraey Y;2022	LEISH	KSA	Other	HB	subnational	all age groups	both
Amin TT et al;2013	LEISH	KSA	CS	CB	subnational	all age groups	both
Asmaa Q et al;2017	LEISH	Yemen	CS	CB	subnational	all age groups	both
Asmae H et al;2014	LEISH	Morocco	CS	CB	subnational	NA/NS	both

First author; year	Disease	Country	Study types	Study setting	Data level	Age group	Sex
Ayaz, S. et al;2011	LEISH	Pakistan	MY C/S	CB	subnational	all age groups	both
Bellali, H. et al;2015	LEISH	Tunisia	MY C/S	Mx	subnational	all age groups	NA/NS
Ben Helel K et al;2017	LEISH	Tunisia	CC	HB	subnational	children/<18	both
Bennis I et al;2015	LEISH	Morocco	MY C/S	CB	subnational	all age groups	both
Doroodgar, A. et al;2012	LEISH	Iran	CS	CB	subnational	all age groups	both
Echchakery M et al;2018	LEISH	Morocco	CS	HB	subnational	non-elderly adults	both
El Hamouchi A et al;2019	LEISH	Morocco	CS	CB	subnational	all age groups	both
Elmekki, M.A. et al;2017	LEISH	KSA	CS	HB	subnational	all age groups	both
Fakoorziba, M.R. et al;2011	LEISH	Iran	CS	CB	subnational	all age groups	NA/NS
Faraj, C. et al;2016	LEISH	Morocco	INT	CB	subnational	NA/NS	NA/NS
Feiz-Haddad, M.-H. et al;2015	LEISH	Iran	CS	HB	subnational	all age groups	both
H., Mahmoudvand et al;2011	LEISH	Iran	CS	CB	subnational	all age groups	both
Haddad, M.H.F. et al;2016	LEISH	Iran	CS	HB	subnational	all age groups	both
Hamzavi, Y. et al;2012	LEISH	Iran	CS	CB	subnational	non-elderly adults	both
Haouas, N. et al;2015	LEISH	KSA	CS	HB	subnational	all age groups	both
Hassanpour, K. et al;2014	LEISH	Iran	CS	HB	subnational	NA/NS	NA/NS
Hayat, M. et al;2013	LEISH	Pakistan	MY C/S	CB	subnational	all age groups	both
Heidari, A. et al;2015	LEISH	Iran	CS	CB	subnational	children/<18	both
Hussain M et al;2017	LEISH	Pakistan	CS	CB	subnational	all age groups	both
Ihsanullah M et al;2021	LEISH	Pakistan	CS	CB	subnational	all age groups	both
Jorjani, O. et al;2019	LEISH	Iran	MY C/S	HB	subnational	all age groups	both
Kanani K et al;2019	LEISH	Jordan	Other	CB	national	all age groups	both
Karami, M. et al;2013	LEISH	Iran	MY C/S	HB	subnational	all age groups	both
Kassiri, H. et al;2012	LEISH	Iran	CS	Mx	subnational	all age groups	both
Kassiri, H. et al;2014	LEISH	Iran	MY C/S	HB	subnational	all age groups	both
Kassiri, H. et al;2019	LEISH	Iran	MY C/S	HB	subnational	all age groups	both
Khademvatan, S. et al;2017	LEISH	Iran	CS	HB	subnational	all age groups	both
Khan A et al;2021	LEISH	Pakistan	MY C/S	CB	subnational	all age groups	both

First author; year	Disease	Country	Study types	Study setting	Data level	Age group	Sex
Khatri ML et al;2016	LEISH	Yemen	C	HB	subnational	all age groups	both
Khazaei, S. et al;2015	LEISH	Iran	CS	HB	subnational	all age groups	both
Khodadadi, S. et al;2012	LEISH	Iran	CS	HB	subnational	all age groups	both
Khosravani, M. et al;2016	LEISH	Iran	CS	HB	subnational	all age groups	both
Khosravani, M. et al;2016	LEISH	Iran	CS	HB	subnational	all age groups	both
M.R., Shirzadi et al;2015	LEISH	Iran	CS	HB	subnational	all age groups	both
Moein, D. et al;2018	LEISH	Iran	MY C/S	HB	subnational	all age groups	both
Mogalli NM et al;2016	LEISH	Yemen	CS	Mx	subnational	all age groups	both
Mohammadi KA et al;2014	LEISH	KSA	CS	CB	subnational	all age groups	both
Mohammadi, J. et al;2022	LEISH	Iran	CS	CB	subnational	all age groups	both
Mohammed MA et al;2018	LEISH	Sudan	CS	CB	subnational	all age groups	both
Mohebali, M. et al;2011	LEISH	Iran	CS	CB	subnational	all age groups	both
Mollalo, A. et al;2014	LEISH	Iran	MY C/S	CB	subnational	NA/NS	NA/NS
Mueller YK et al;2012	LEISH	Sudan	MY C/S	CB	subnational	all age groups	both
Nackers, F. et al;2015	LEISH	Sudan	CC	CB	subnational	all age groups	both
Nassar AA et al;2021	LEISH	Yemen	CC	CB	subnational	all age groups	both
Nateghi Rostami, M. et al;2013	LEISH	Iran	CS	HB	subnational	all age groups	both
Qureshi NA et al;2016	LEISH	Pakistan	CS	CB	subnational	children/<18	male
Sadeq M;2016	LEISH	Morocco	CS	CB	national	all age groups	both
Sakhaei, S. et al;2019	LEISH	Iran	MY C/S	HB	subnational	all age groups	both
Saki, J. et al;2016	LEISH	Iran	CS	HB	subnational	all age groups	both
Salih, O. et al;2020	LEISH	Sudan	MY C/S	HB	subnational	all age groups	both
Sarkari, B. et al;2015	LEISH	Iran	CS	CB	subnational	all age groups	both
Sarkari, B. et al;2016	LEISH	Iran	CS	HB	subnational	all age groups	both
Sherba, A.Z. et al;2018	LEISH	Iraq	CS	HB	subnational	all age groups	both
Shirzadi, M.R. et al;2015	LEISH	Iran	C	HB	subnational	NA/NS	NA/NS
Soltani, S. et al;2019	LEISH	Iran	MY C/S	HB	subnational	all age groups	both
Soomro, A.A. et al;2012	LEISH	Pakistan	MY C/S	HB	subnational	all age groups	both

First author; year	Disease	Country	Study types	Study setting	Data level	Age group	Sex
Tofighi Naeem, A. et al;2014	LEISH	Iran	MY C/S	HB	subnational	children/<18	both
Ullah K et al;2016	LEISH	Pakistan	Other	HB	subnational	all age groups	both
van den Bogaart E et al;2013	LEISH	Sudan	CC	HB	subnational	all age groups	both
Yaghoobi-Ershadi, M.R. et al;2013	LEISH	Iran	CS	CB	subnational	all age groups	both
Youssef, A. et al;2019	LEISH	Syrian Arab [illegible]	MY C/S	HB	subnational	all age groups	both
Zahra, N. et al;2018	LEISH	Iran	MY C/S	HB	subnational	all age groups	both
Zeb I et al;2020	LEISH	Pakistan	CS	CB	subnational	children/<18	male
Zeb I et al;2021	LEISH	Pakistan	MY C/S	CB	subnational	all age groups	both
Alavi, S.M. et al;2014	LEPT	Iran	MY C/S	Mx	subnational	all age groups	both
El Azhari, M. et al;2020	LEPT	Morocco	CS	Mx	subnational	all age groups	both
Esmaeili, S. et al;2017	LEPT	Iran	MY C/S	CB	subnational	all age groups	both
Majd, N.S. et al;2012	LEPT	Iran	MY C/S	HB	subnational	all age groups	NA/NS
Sahneh, E. et al;2019	LEPT	Iran	CC	HB	subnational	all age groups	both
Sakhaee, E. et al;2011	LEPT	Iran	MY C/S	HB	subnational	NA/NS	NA/NS
Adegboye, O. et al;2019	MERS	KSA	MY C/S	Mx	national	all age groups	both
Adegboye, O.A. et al;2017	MERS	KSA	CS	Mx	subnational	all age groups	both
Al Ghamdi M et al;2016	MERS	KSA	CS	HB	subnational	all age groups	both
Al Sulayyim, H.J. et al;2020	MERS	KSA	CS	HB	subnational	all age groups	both
Al-Hameed, F. et al;2016	MERS	KSA	C	HB	subnational	all age groups	both
Al-Raddadi, R.M. et al;2020	MERS	KSA	CS	HB	national	all age groups	both
Aleanizy FS et al;2017	MERS	KSA	CS	HB	national	all age groups	both
Alfaraj, S.H. et al;2019	MERS	KSA	C	HB	subnational	all age groups	both
Alghamdi, I.G. et al;2014	MERS	KSA	MY C/S	Mx	subnational	all age groups	both
Alhamlan FS et al;2017	MERS	KSA	MY C/S	Mx	national	all age groups	both
Alqahtani, F.Y. et al;2019	MERS	KSA	MY C/S	HB	national	all age groups	both
Alraddadi BM et al;2016	MERS	KSA	CC	HB	subnational	all age groups	both
Alraddadi BM et al;2016	MERS	KSA	C	HB	subnational	all age groups	both
Alsharif, K.F. et al;2021	MERS	KSA	CS	HB	subnational	all age groups	both

First author; year	Disease	Country	Study types	Study setting	Data level	Age group	Sex
Altamimi, A. et al;2020	MERS	KSA	MY C/S	HB	subnational	all age groups	both
Altamimi, A. et al;2020	MERS	KSA	CS	Mx	subnational	all age groups	both
Arabi, Y.M. et al;2014	MERS	KSA	CS	HB	subnational	all age groups	both
Arwady, M.A. et al;2016	MERS	KSA	Other	Mx	subnational	all age groups	both
Barry, M. et al;2020	MERS	KSA	Other	HB	subnational	all age groups	both
Ben Abid, F. et al;2021	MERS	Qatar	CS	HB	subnational	all age groups	both
Ebrahim, S.H. et al;2021	MERS	KSA	CS	HB	national	all age groups	both
Feikin, D.R. et al;2015	MERS	KSA	CS	HB	subnational	all age groups	both
Garout, M.A. et al;2018	MERS	KSA	CS	HB	subnational	all age groups	both
H.E., El Bushra et al;2016	MERS	KSA	CS	HB	subnational	all age groups	both
Habib, A.M.G. et al;2019	MERS	KSA	CS	HB	subnational	all age groups	both
Hastings DL et al;2016	MERS	KSA	CS	HB	subnational	all age groups	both
Jose J et al;2021	MERS	KSA	C	HB	subnational	all adults	both
Khudhair, A. et al;2019	MERS	UAE	MY C/S	CB	subnational	non-elderly adults	both
M√°ller MA et al;2015	MERS	KSA	CS	Mx	national	all age groups	both
Noorwali AA et al;2015	MERS	KSA	CS	HB	subnational	all age groups	both
Oboho, I.K. et al;2015	MERS	KSA	Other	Mx	subnational	all age groups	both
Saad, M. et al;2014	MERS	KSA	CS	HB	subnational	all age groups	both
Shalhoub S et al;2018	MERS	KSA	C	HB	national	non-elderly adults	both
Sherbini, N. et al;2017	MERS	KSA	CS	HB	subnational	all age groups	both
Sikkema, R.S. et al;2017	MERS	Qatar	CC	HB	subnational	non-elderly adults	both
Abdulmoghni, R.T. et al;2021	Rabies	Yemen	LS	CB	national	all age groups	both
Al Abaidani, I. et al;2015	Rabies	Oman	Other	HB	national	all age groups	both
Ali, M.I. et al;2021	Rabies	Pakistan	MY C/S	HB	subnational	all age groups	both
Bennasrallah, C. et al;2021	Rabies	Tunisia	Other	HB	subnational	all age groups	both
Dehghani, A. et al;2019	Rabies	Iran	MY C/S	HB	subnational	all age groups	both
Kalthoum S et al;2021	Rabies	Tunisia	LS	HB	national	all age groups	both
Muhammad Aslam, Bajwa et al;2012	Rabies	Pakistan	MY C/S	CB	subnational	all age groups	both

First author; year	Disease	Country	Study types	Study setting	Data level	Age group	Sex
Abuelyazeed A., Elsheikh et al;2011	RVF	KSA	MY C/S	HB	subnational	NA/NS	NA/NS
Ahmed, A. et al;2020	RVF	Sudan	MY C/S	CB	subnational	all age groups	both
Al Azraqi TA et al;2013	RVF	KSA	CS	HB	subnational	children/<18	both
Al-Azraqi TA et al;2012	RVF	KSA	MY C/S	HB	subnational	all age groups	both
Almasri, M. et al;2019	RVF	KSA	Other	CB	subnational	all age groups	NA/NS
Andayi, F. et al;2014	RVF	Djibouti	CS	CB	subnational	all age groups	both
Bashir, R.S.E. et al;2019	RVF	Sudan	Other	CB	subnational	NA/NS	NA/NS
Baudin M et al;2016	RVF	Sudan	MY C/S	HB	subnational	all adults	female
Memish, Z.A. et al;2015	RVF	KSA	MY C/S	CB	subnational	all adults	both
Mohamed N et al;2019	RVF	Sudan	MY C/S	HB	subnational	all age groups	both
Zayed, A.B. et al;2015	RVF	Egypt	Other	CB	subnational	NA/NS	NA/NS
Chinikar, S. et al;2012	WNF	Iran	MY C/S	HB	subnational	all age groups	both
Niazi SK et al;2017	WNF	Pakistan	MY C/S	HB	subnational	all adults	both
Riabi S et al;2014	WNF	Tunisia	MY C/S	HB	subnational	all age groups	both
Shaibi, T. et al;2017	WNF	Tunisia	MY C/S	HB	subnational	all adults	both
Soghaier MA et al;2013	YF	Sudan	CS	CB	subnational	non-elderly adults	both

Note: CCHF— Crimean-Congo hemorrhagic fever; CHIK— Chikungunya; DF—Dengue Fever; MERS— Middle East Respiratory Syndrome; ZIA— Zoonotic Influenza A; LEISH— Leishmaniasis; LEP—Leptospirosis; RAB—Rabies; RVF—Rift Valley Fever; WNF—West Nile Fever; YF—Yellow Fever; NA/NS— Not available/specified; CS— cross-sectional study; MY CS— multi-year cross-sectional study; LS— longitudinal study; C— cohort; CC— case control; INT— interventional'; KSA— Kingdom of Saudi Arabia; UAE— United Arab Emirates; HB— Hospital based; CB— Community based; Mx—mixed
